# Neuronal SAM68 differentially regulates alternative last exon splicing and ensures proper synapse development and function

**DOI:** 10.1016/j.jbc.2023.105168

**Published:** 2023-08-16

**Authors:** Mohamed Darwish, Masatoshi Ito, Yoko Iijima, Akinori Takase, Noriko Ayukawa, Satoko Suzuki, Masami Tanaka, Kanae Komori, Daisuke Kaida, Takatoshi Iijima

**Affiliations:** 1 Division of Basic Medical Science and Molecular Medicine, Department of Molecular Life Science, School of Medicine, Tokai University, Kanagawa, Japan; 2 Department of Biochemistry, Faculty of Pharmacy, Cairo University, Cairo, Egypt; 3 The Support Center for Medical Research and Education, Tokai University, Kanagawa, Japan; 4 Tokai University Institute of Innovative Science and Technology, Isehara, Kanagawa, Japan; 5 Graduate School of Medicine and Pharmaceutical Sciences, University of Toyama, Toyama, Japan

**Keywords:** alternative splicing, ALE, 3′UTR, SAM68, synapse, PCDH15, PAS, RNA-binding protein, gamma-aminobutyric acid (GABA), neurodevelopment

## Abstract

Alternative splicing in the 3′UTR of mammalian genes plays a crucial role in diverse biological processes, including cell differentiation and development. SAM68 is a key splicing regulator that controls the diversity of 3′UTR isoforms through alternative last exon (ALE) selection. However, the tissue/cell type-specific mechanisms underlying the splicing control at the 3′ end and its functional significance remain unclear. Here, we show that SAM68 regulates ALE splicing in a dose-dependent manner and the neuronal splicing is differentially regulated depending on the characteristics of the target transcript. Specifically, we found that SAM68 regulates interleukin-1 receptor-associated protein splicing through the interaction with U1 small nuclear ribonucleoprotein. In contrast, the ALE splicing of protocadherin-15 (*Pcdh15*), a gene implicated in several neuropsychiatric disorders, is independent of U1 small nuclear ribonucleoprotein but modulated by the calcium/calmodulin-dependent protein kinase signaling pathway. We found that the aberrant ALE selection of *Pcdh15* led to a conversion from a membrane-bound to a soluble isoform and consequently disrupted its localization into excitatory and inhibitory synapses. Notably, the neuronal expression of the soluble form of PCDH15 preferentially affected the number of inhibitory synapses. Moreover, the soluble form of PCDH15 interacted physically with α-neurexins and further disrupted neuroligin-2-induced inhibitory synapses in artificial synapse formation assays. Our findings provide novel insights into the role of neuron-specific alternative 3′UTR isoform selections in synapse development.

Thousands of mammalian genes encode alternatively spliced isoforms in their 3′UTR. Alternative 3′UTR isoforms are generated through alternative last exon (ALE) splicing and alternative polyadenylation (1, 2). Most human genes have multiple ALEs and polyadenylation sites (PASs), which enable the expression of distinct 3′UTR isoforms in a tissue- or cell-type-dependent manner (3). The differences in the 3′UTR through ALE splicing may be linked to the establishment of cell identities (4) and are accompanied by cell development (5, 6). Thus, ALE splicing is dynamically regulated in a spatiotemporal manner and is highly implicated in human diseases including hematological, immunological, and neurological diseases and cancer (7). Transcriptomic diversity through the spatiotemporal and dynamic regulation of the ALE contributes to biological processes such as cell differentiation and identification. Information in the 3′UTR regulates mRNA targeting, translational efficiency, and stability (8). In neurons, specific mRNAs are localized near synapses, where localization is regulated by ALE splicing (6). This regulation of local translation allows neurons to deal rapidly with synaptic functions and underlies important cellular processes such as dendrite arborization, synapse formation, and axon guidance (9). ALE selection can change protein isoforms by truncating the coding sequence and/or providing alternative carboxy-terminal extensions of the protein, which dynamically change its functions (10). The usage of the ALE may underlie functional diversity at the transcriptomic and proteomic levels. However, the tissue- and/or cell-type-specific mechanisms underlying the control of ALE splicing in the nervous system and their functional significance remain unclear.

Sarcoma (Src) associated in mitosis (SAM68; 68 kDa) is a K-homology domain RNA-binding protein that belongs to the signal transduction and activation of RNA family. SAM68 is a tissue-specific key regulator of the diversity of neuronal 3′UTR isoforms through ALE splicing as the KO of *Sam68* preferentially causes premature termination at internal PASs (10, 11, 12). SAM68 synergistically acts with U1 small nuclear ribonucleoprotein (U1 snRNP) on pre-mRNAs to prevent improper termination in specific tissues (11, 13) by preventing cleavage and polyadenylation at cryptic PASs through a process called “telescripting” (14). The interaction between SAM68 and U1 snRNP ensures proper 3′ processing during germ cell differentiation (11). Thus, the mechanisms by which SAM68 regulates ALE and its subsequent effects on neuronal function are beginning to be uncovered.

We previously found that SAM68 regulates the selection of ALEs in several genes, including *protocadherin-15* (*Pcdh15*) and interleukin 1-receptor accessory protein (*Il1rap*) (10). PCDH15 is a nonclustered protocadherin that plays an essential role in the maintenance of retinal and cochlear function (15). The gene is responsible for Usher syndrome, which presents with hearing loss following retinal degeneration (16, 17). Additionally, *Pcdh15* gene variations, such as copy number variations and single nucleotide polymorphisms, have been implicated in neurodevelopmental disorders such as autism, bipolar disorder, and schizophrenia (18, 19, 20, 21). In this study, we aimed to elucidate the mechanism by which neuronal SAM68 regulates ALE splicing of the target transcripts and its relevant significance on neuron function. We revealed that the neuronal SAM68 differentially regulates ALE splicing depending on the characteristics of the target transcript. Specifically, we observed that ALE splicing of *Pcdh15* but not *Il1rap* is modulated by neuronal activity and calcium/calmodulin-dependent protein kinase (CaMK) signaling. Moreover, the aberrant ALE splicing of *Pcdh15* perturbs synapse formation and function through the disruption of proper protein localization into excitatory and inhibitory synapses. Together, these data raise the possibility that the aberrant ALE splicing of *Pcdh15* may be associated with neurological disorders.

## Results

### SAM68 is a dose-dependent ALE splicing regulator of *Pcdh15* and *Il1rap*

**Figure 1:**
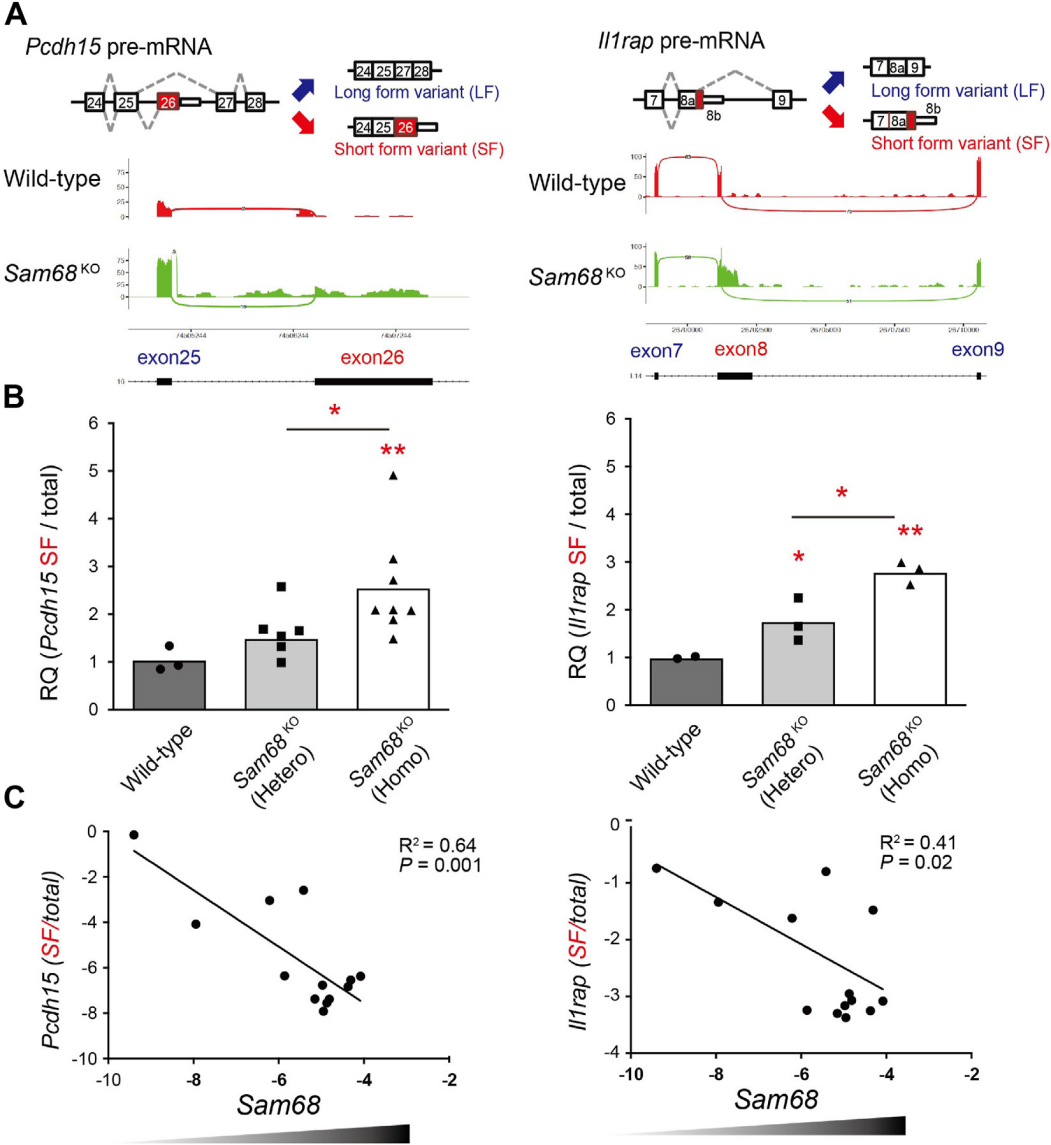
**SAM68 is a dose-dependent ALE splicing regulator of *Pcdh15* and *Il1ra****p*. *A*, aberrant 3′UTR exon selection of the representative genes in *Sam*68^KO^ brains. The *upper panels* show a schematic illustration of the selection of the ALE in two representative genes targeted by SAM68, including *Pcdh15* (exon 26) and *Il1rap* (exon 8). The *white rectangle* represents the 3′ UTR of the short form variants. *Lower panels* show sashimi plots of the ALE events in the relevant genes. Each plot includes cassette exons and intron retentions. The *red plots* represent the WT mice, and the *green plots* represent *Sam*68^KO^. The *X*-axes show genomic loci, and the *Y*-axes indicate transcription intensity. A “sashimi-like” region in each plot indicates an exon region, and the blank regions between them indicate intronic regions. The numbers on the bridges crossing exons indicate junction reads. The raw data [the DDBJ database (6781), with the accession number of DRA6781, with bioproject accession number of PRJDB6781] was obtained in the previous study (10). The RNA-seq was based on the UCSC genome browser Mouse NCBI37/mm10 assembly. *B*, relative expression of the short-form (SF) variants of *Pcdh15* and *Il1rap* from the WT, *Sam68*^KO^ (hetero), and *Sam68*^KO^ (homo) brains. The Ct values of the SF transcripts were normalized to those of the total transcripts. n = 3 to 7 experiments per condition. One-way ANOVA was followed by Tukey’s multiple comparisons test. *C*, correlation between *Sam68* transcript levels and SF variants of *Il1rap* and *Pcdh15* from various brain regions and tissues. CT values of SF transcripts were normalized to those of the total transcripts and the Ct values of *Sam68* were normalized to those of *Gapdh*. The correlation coefficient (R^2^) and significance level between *Sam68* and the SF transcripts are shown in the scatter plot. ALE, alternative last exon; pcdh15, protocadherin-15; Il1rap, interleukin 1-receptor accessory protein; UCSC, University of California, Santa Cruz.

We previously demonstrated that SAM68 regulates ALE splicing for several neuronal targets such as *Pcdh15*, *Il1rap*, ceruloplasmin, and leucine-rich repeated coiled-coil protein 1 (10). These genes contain proximal 3′ UTR cryptic exons (exon 26 for *Pcdh15*, exon 8b for *Il1rap*, exon 18 for ceruloplasmin, and exon 19 for leucine-rich repeated coiled-coil protein *11*), which undergo skipping in the presence of SAM68, resulting in predominant expression of long isoforms. RNA sequencing data revealed a pronounced inclusion of these proximal 3′ UTR cryptic exons in the brains of SAM68-deficient mice, leading to a switch from long to short isoforms through ALE splicing (Figs. 1*A* and S1, *A* and *B*).

To evaluate the impact of SAM68 dosage on the targeted transcripts in different genotypes and tissues, we measured the short isoforms as a representative of cryptic exon inclusion. The changes in the short isoforms exhibited higher sensitivity and greater detectability in our experiments, given the predominant expression of the long isoform in mouse tissue. Interestingly, we observed an inverse correlation between the ratios of the short isoforms (including cryptic exons) of the *Pcdh15* and *Il1rap* and the genetic and expression doses of *Sam68* in mice (Fig. 1, *B* and *C*). Taken together, our data strongly support the role of SAM68 as a dose-dependent regulator of ALE splicing for *Pcdh15* and *Il1rap*.

### Neuronal SAM68 interacts with U1 snRNP to regulate ALE splicing of *Il1rap* but not *Pcdh15*

**Figure 2:**
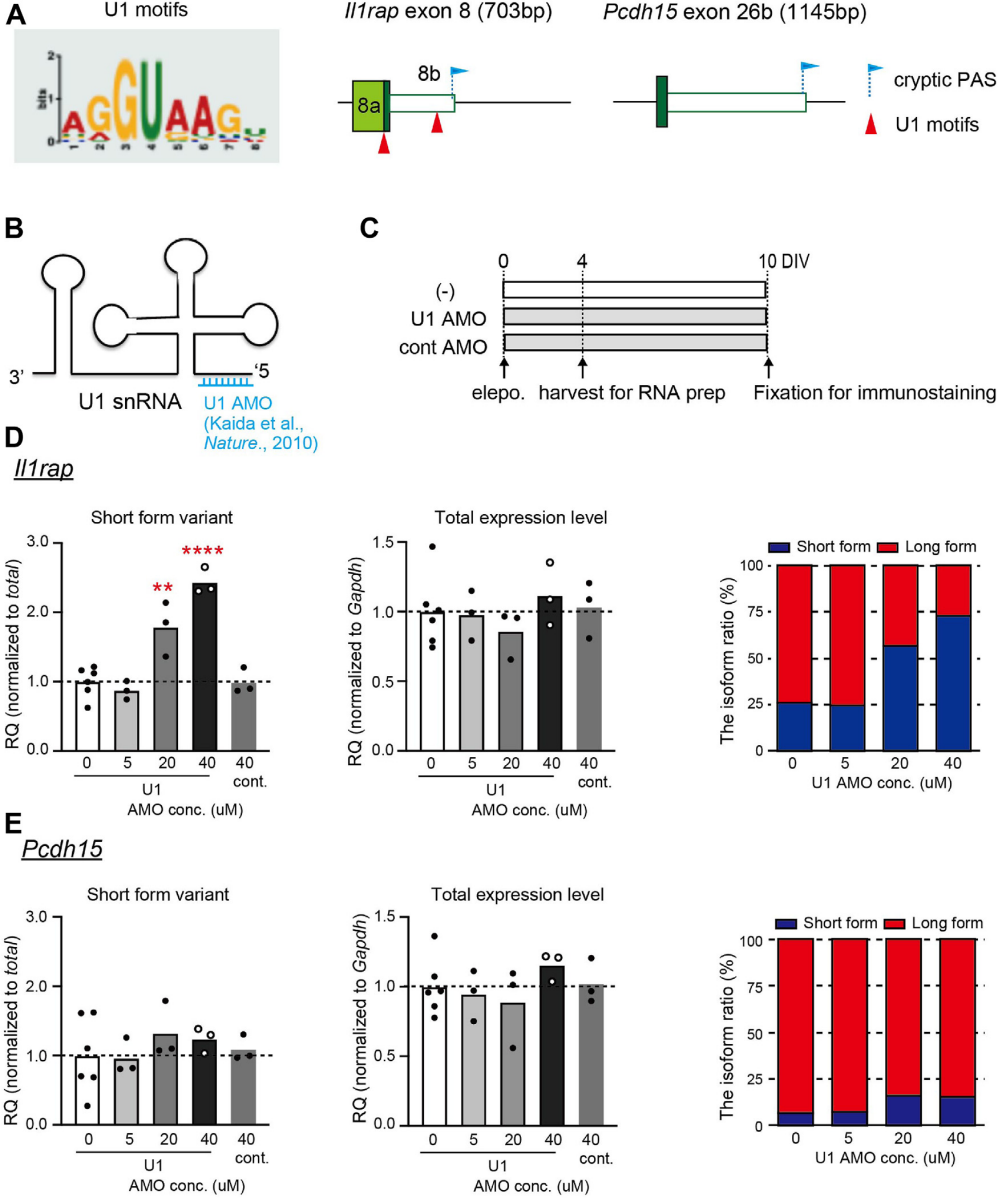
**Distinct usage of U1 snRNP on ALE isoform selections of SAM68-targeted transcripts.***A*, logo representing the U1-binding consensus sequence (U1 motif) scanned for motif analysis enrichment (*left*). Schematic illustration showing the candidate U1 motif positions around ALE sequences in *Il1rap* (*middle*) and *Pcdh15* (*right*). U1 motifs and the cryptic polyadenylation sites (PAS) of each exon are labeled in arrowheads and flags, respectively. *B*, schematic representation of U1 snRNP. The antisense morpholino oligonucleotide binding site at the 5′ end is shown in *blue* (U1 AMO). *C*, schematic overview showing the protocol used for the U1 AMO-treated cortical neuronal culture. U1 or control AMOs were electroporated into plated cells at DIV0. The cells were harvested at DIV4 for RT-qPCR or further maintained until DIV10 for immunostaining. *D* and *E*, relative expression of the total mRNA and short-form (SF) variants of *Il1rap* (*D*) and *Pcdh15* (*E*), and the abundance ratio of the SF (*red*) to LF (*blue*) in cultured cortical neurons treated with different concentrations of U1 AMOs by RT-qPCR. The Ct values of the total transcripts were normalized to those of *Gapdh*, and the Ct value of each alternative isoform was normalized to the total transcripts. n = 3 to 6 experiments per condition. One-way ANOVA was followed by Tukey’s multiple comparisons test. (*D*) *Il1rap.* Total expression and SF variant. (*E*) *Pcdh15.* Total expression and SF variant. ALE, alternative last exon; AMOs, antisense morpholino oligonucleotide; Il1rap, interleukin 1-receptor accessory protein; pcdh15, protocadherin-15; RT-qPCR, reverse transcription-quantitative PCR; U1 snRNP, U1 small nuclear ribonucleoprotein.

To elucidate the mechanism by which neuronal SAM68 regulates ALE splicing, we investigated whether the splicing of SAM68-targeted transcripts is regulated by interactions with U1 snRNP in cultured neurons (11, 13). U1 snRNP binds to multiple intronic sites near cryptic PASs and prevents premature termination on a genome-wide scale (14, 22). We identified U1 motif-like sequences at genomic loci around the cryptic PASs of *Il1rap* but not in the vicinity of exon 26 of *Pcdh15* (Figs. 2*A* and S2*A*). Subsequently, we examined the impact of functional knockdown of U1 snRNP on ALE selection of *Il1rap* and *Pcdh15* using U1 antisense morpholino oligonucleotides (AMOs) that covers the 5′ end of U1 snRNA, as reported previously (14) (Fig. 2*B*). U1 AMOs and control AMOs were electroporated at different concentrations into primary cortical cultures followed by quantitative PCR (qPCR) assessment of ALE selection (Fig. 2*C*). We confirmed that U1 AMO had no significant impact on *Sam68* expression (Fig. S2*C*). In U1 AMO-treated neurons, there was a significant concentration-dependent increase in the short-form (SF) variant of *Il1rap*, accompanied by reductions in its long-form (LF) variants (Figs. 2*D* and S2*B*). Notably, the total amount of *Il1rap* remains unchanged, indicating that the observed alteration was due to a shift in long-to-short isoform conversion. In contrast to *Il1rap*, the *Pcdh15* variants were not significantly altered in response to the knockdown of U1 snRNP (Figs. 2*E* and S2*B*). Additionally, we examined the numbers of neurons and synapses in the absence and presence of U1 AMOs. We found a significant difference in the number of synapses, but not in the number of neurons (Fig. S2, *D*–*F*), indicating that U1 AMO electroporation may influence neuronal differentiation and/or maturation without causing lethality in neuron cultures under these conditions. Thus, our results suggest differential utilization of U1 snRNP between targeted transcripts.

### SAM68 regulates neuronal activity-dependent ALE isoform selections of *Pcdh15*

**Figure 3:**
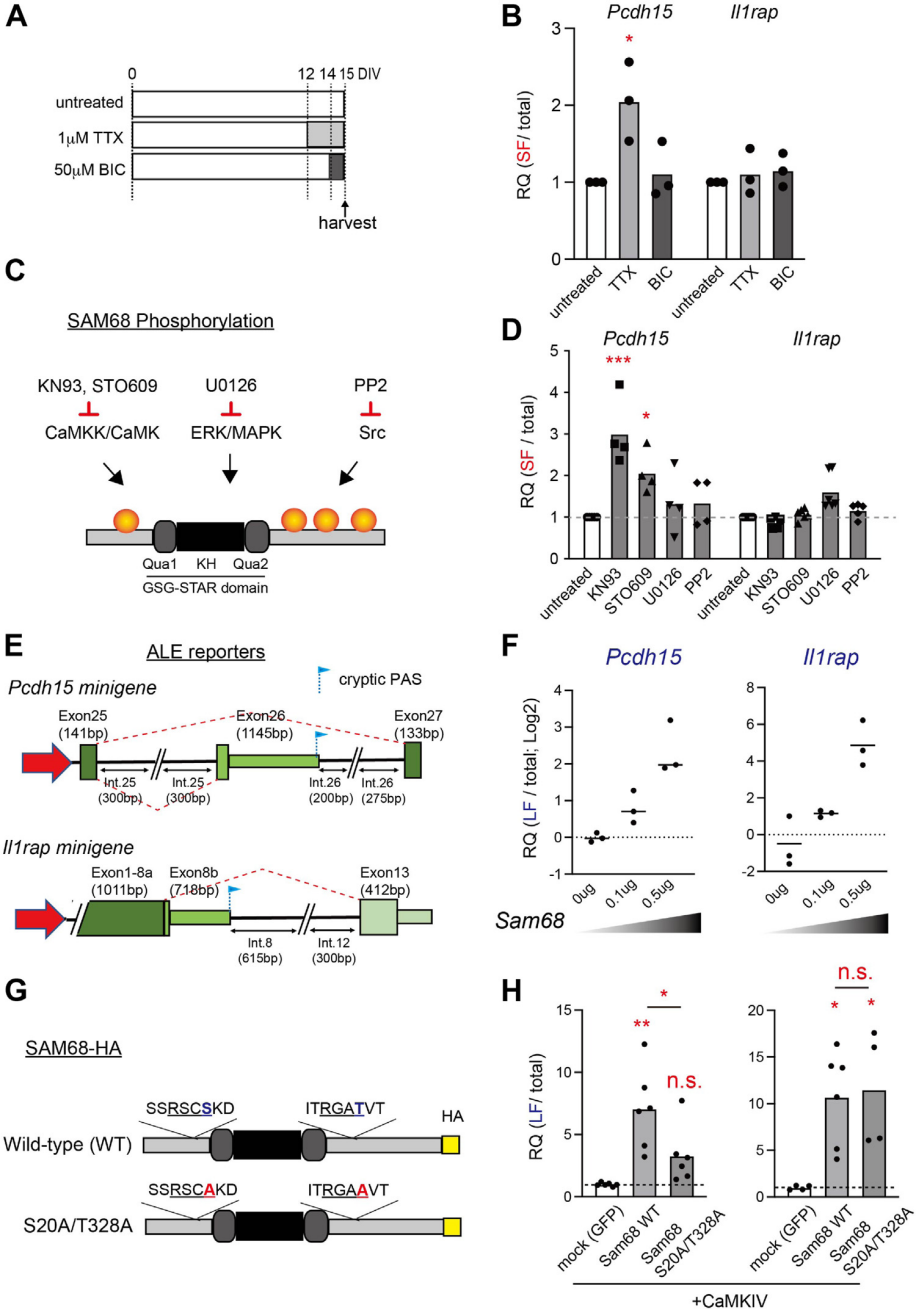
**Distinct regulation of SAM68-regulated ALE splicing by neuronal activity and cellular signals**. *A*, schematic overview of the pharmacological treatment protocol. Cultured cortical neurons were treated with bicuculine (BIC) (50 μM for 1 day) and tetrodotoxin (TTX) (1 μM for 3 days) before harvesting on DIV15. *B*, relative expression of short-form (SF) variants of *Pcdh15* and *Il1rap*. Cultured cortical neurons were treated with TTX and BIC as shown in (*A*). The Ct values of the SF transcripts were normalized to those of the total transcripts. n = 3 experiments per condition. One-way ANOVA was followed by Tukey’s multiple comparisons test. *C*, schematic illustration showing SAM68 phosphorylation by several cellular signals/kinases and their inhibitors (5 μM KN93, 10 μM STO609, 5 μM U0126, or 10 μM PP2). *D*, relative expression of the SF variants of *Pcdh15* and *Il1rap*. Cultured cortical neurons were treated with kinase inhibitors are shown in (*C*) on DIV14 and harvested on DIV15. The Ct values of the SF transcripts were normalized to those of the total transcripts. n = 4 to 6 experiments per condition. One-way ANOVA was followed by Tukey’s multiple comparisons test. *E*, schematic diagram of ALE reporters for *Pcdh15* (*upper*) and *Il1rap* (*lower*). The *Pcdh15* ALE reporter construct contains a sequence with constitutive exons 25 and 27 (*dark green*), and cryptic ALE 26 (*light green*). The *Il1rap* ALE reporter construct is a chimeric sequence with constitutive exons 8a (*dark green*) and alternative ALEs 8b and 13 (*light green*). Introns are shown as *lines*. The sizes of introns and exons are indicated (drawings not to scale). *F*, relative expression of the long-form (LF) variants of *Pcdh15* and *Il1rap* when ALE reporter vectors were cotransfected into HEK293T cells with different doses of SAM68 expression vector. The Ct values of the LF transcripts were normalized to those of the total transcripts. *G*, schematic illustration of SAM68 WT and mutant (S20A/T328A) for CaMKIV recognition motif. The position of the recognition motifs (R-X-X-S/T) is *underlined*. Serine and threonine (*blue*) were point mutated to alanine (*red*). *H*, relative expression of the LF variants of *Pcdh15* and *Il1rap* when ALE reporter vectors were cotransfected into HEK293T cells with SAM68 WT or phospho-mutant (S20A/T328A) in the presence of CaMKIV. The Ct values of the LF transcripts were normalized to those of the total transcripts. n = 4 to 6 experiments per condition. One-way ANOVA was followed by Tukey’s multiple comparisons test. ALE, alternative last exon; CaMK, calcium/calmodulin-dependent protein kinase; Il1rap, interleukin 1-receptor accessory protein; pcdh15, protocadherin-15.

We next investigated other SAM68-dependent mechanisms that regulate ALE splicing of the target transcripts. Our previous study has demonstrated that SAM68 regulates neuronal activity-dependent alternative splicing (23). Therefore, we investigated whether SAM68-mediated ALE splicing could be influenced by neuronal activity. To assess this, we treated differentiated cortical neuron cultures with bicuculine or tetrodotoxin (TTX) to increase or decrease neuronal activity, respectively (Fig. 3*A*). Interestingly, we observed a significant increase in the SF ratio of *Pcdh15* following TTX treatment (Fig. 3*B*). In contrast, the isoform ratios of *Il1r*ap were not significantly altered by either treatment (Fig. 3*B*). These results suggest that the ALE of *Pcdh15* but not *Il1rap* is influenced by spontaneous neuronal activity. To get insight into the signaling pathways that underlie neuronal activity-mediated ALE splicing of SAM68 targets, we performed pharmacological inhibition of SAM68-modifying kinases known to be activated following neuronal activity. Specifically, CaMK, extracellular signal-regulated kinase/mitogen-activated protein kinase, and Src kinase have been reported to phosphorylate SAM68 in several sites that modulate its splicing activity (23, 24, 25) (Fig. 3*C*). Remarkably, the short isoform ratio of *Pcdh15* was significantly increased by CaMK inhibitors, KN93, and STO609, while there were no significant changes in response to extracellular signal-regulated kinase/mitogen-activated protein kinase inhibitor, U0126, or the Src inhibitor, PP2 (Fig. 3*D*). In contrast, the ALE splicing of *Il1rap* did not significantly change by the actions of any of these inhibitors.

Given that CaMK inhibitors can affect multiple targets other than SAM68, we aimed to investigate whether CaMK-mediated phosphorylation of SAM68 specifically modulates the ALE skipping of *Pcdh15*. To address this, we constructed *Pcdh15* and *Il1rap* minigene splice reporters to test whether SAM68 and its variants properly perform the cryptic ALE splicing. These splice reporters spanned the cryptic exons *Pcdh15* exon 26 and *Il1rap* exon 8b, respectively (Fig. 3*E*). Upon transfection into HEK293T cells, those cryptic exons are included which allows us to evaluate whether SAM68 promotes the skipping of these cryptic ALE exons. In contrast to mouse tissues, the short isoforms with cryptic exons are predominant in HEK293T cells; therefore, we measured the long isoforms as representative of cryptic exons skipping. Consistent with our findings in mouse tissues (Fig. 1, *B* and *C*), we observed that transfected SAM68 effectively skipped cryptic exons in a dose-dependent manner (Fig. 3*F*). These results indicate that the minigene reporters robustly respond to SAM68 and serve as valuable tools for investigating the impact of SAM68 variants on ALE splicing. Furthermore, we constructed a phospho-mutant SAM68 (S20A and T328A) plasmid in which serine 20 and threonine 328 were replaced with alanine residues to impede phosphorylation by CaMKIV (Fig. 3*G*). We previously identified serine 20 of SAM68 is phosphorylated by CaMKIV upon depolarization (23) and threonine 328 as a putative site for CaMKIV phosphorylation as it conforms to the recognition motif of CaMKIV (R-X-X-S/T). Subsequently, we transfected HEK293T cells with *Pcdh15/Il1rap* ALE minigene reporters along with WT and phospho-mutant (S20A/T328A) SAM68. We found that WT SAM68 significantly increased the skipping of cryptic exons (*Pcdh15* exon 26 in Pcdh15 reporter and *Il1rap* exon 8b in Il1rap reporter), whereas the phospho-mutant SAM68 notably attenuated the skipping of *Pcdh15* exon 26, but not *Il1rap* exon 8b (Fig. 3*H*). These findings suggest that CaMK-mediated signaling, which is triggered by spontaneous neuronal activity, controls the ALE selection of *Pcdh15* by SAM68. Thus, the distinct ALE splicing patterns observed in *Pcdh15* and *Il1rap* imply the existence of at least two SAM68-dependent ALE selection mechanisms in the nervous system.

### Aberrant ALE selection of Pcdh15 alters its subcellular localization in neurons

**Figure 4:**
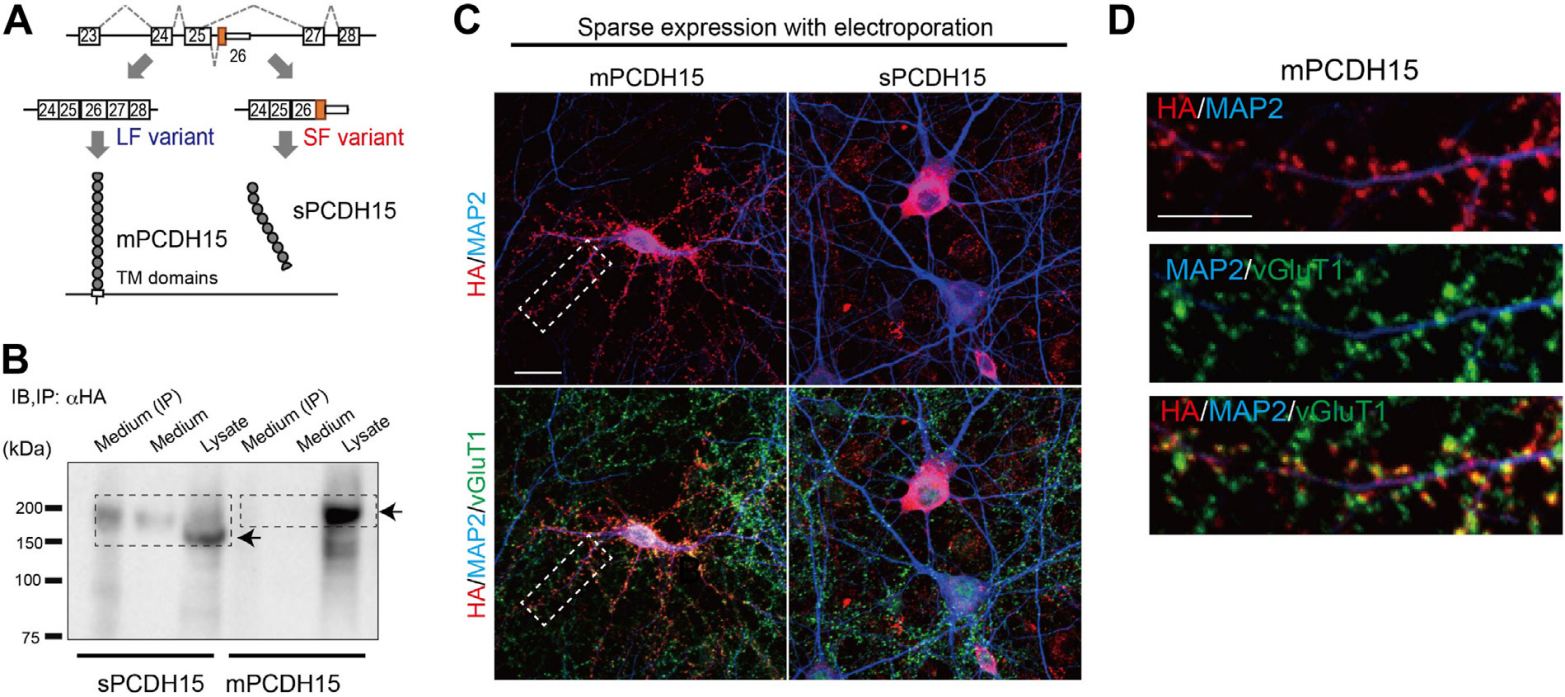
**Aberrant ALE splicing disrupts PCDH15 localization into excitatory postsynapses**. *A*, schematic illustration of the ALE selection of Pcdh15. The insertion of exon 26 causes the truncation of the large coding sequence at the C′ terminal and transmembrane (TM) domain and expresses the soluble form (sPCDH15) instead of the membrane-bound type (mPCDH15). *B*, Western blot showing the detection of sPCDH15 and mPCDH15 in cell lysate and culture medium. HEK293T cells were transfected with sPCDH15-HA or mPCDH15-HA constructs as indicated at the bottom. Cell lysate and supernatant without (*medium*) and with immunoprecipitation (medium IP) were analyzed for the expression of PCDH15 using antibodies to HA. Immunoprecipitation was performed using antibodies to HA. *C*, immunostaining of the subcellular localization of mPCDH15 (*left*) and sPCDH15 (*right*) in cultured cortical neurons (at DIV14) after electroporation with HA-tagged mPCDH15 or sPCDH15 lentiviral vectors at DIV4. VGluT1 and MAP2 stained excitatory synaptic boutons and the overall morphology of neurons, respectively. *D*, high magnification images of the area surrounded by *dashed rectangles* in (*C*). The scale bars represent 10 μm in (*C*) and 5 μm in (*D*). ALE, ALE, alternative last exon; pcdh15, protocadherin-15; HA, hemagglutinin; mPCDH15, membrane-bound form of PCDH15; sPCDH15, soluble form of PCDH15; VGluT1, vesicular glutamate transporter 1.

We next investigated the impact of altered SAM68-dependent ALE splicing on the protein characteristics. We previously found that the altered ALE usage of *Il1rap* in *Sam68* ^KO^ brains resulted in the conversion of its membrane-bound isoform to a secreted form, which results in alterations in *Il1rap* neuronal functions (10). We, therefore, assessed whether the aberrant ALE selection of *Pcdh15* affects its cellular localization. When expressed in HEK293T cells, a membrane-bound form of PCDH15 (mPCDH15) was robustly detected in the cell lysate, while a soluble form of PCDH15 (sPCDH15) was found in both the lysate and medium (Fig. 4*B*). This indicates that the inclusion of exon 26 in *pcdh15* led to the conversion from the mPCDH15 to the sPCDH15 (Fig. 4, *A* and *B*).

To assess the subcellular localization of PCDH15 in cultured neurons, we attempted immunostaining using several antibodies against PCDH15. However, reliable immunoreactivity was not achieved with these antibodies. Therefore, we sparsely expressed mPCDH15 and sPCDH15 tagged with hemagglutinin (HA) at the C′-terminal in cultured cortical neurons and evaluated subcellular localization with an anti-HA antibody. The immunoreactivity of mPCDH15-HA was robustly accumulated on the spine heads of the neurons (Fig. 4*C*). The localization was further confirmed by costaining with an antibody against a vesicular glutamate transporter 1 (VGluT1), an excitatory synaptic marker (Fig. 4*D*), indicating that mPCDH15 is abundantly localized into excitatory postsynaptic neurons. In contrast, most of the sPCDH15-HA were retained in the soma and were not inserted into the postsynapses (Fig. 4*C*). Thus, the ALE selection of *Pcdh15* significantly affected its synapse localization in cortical neurons.

### Aberrant ALE selection of *Pcdh15* influences synapse development

**Figure 5:**
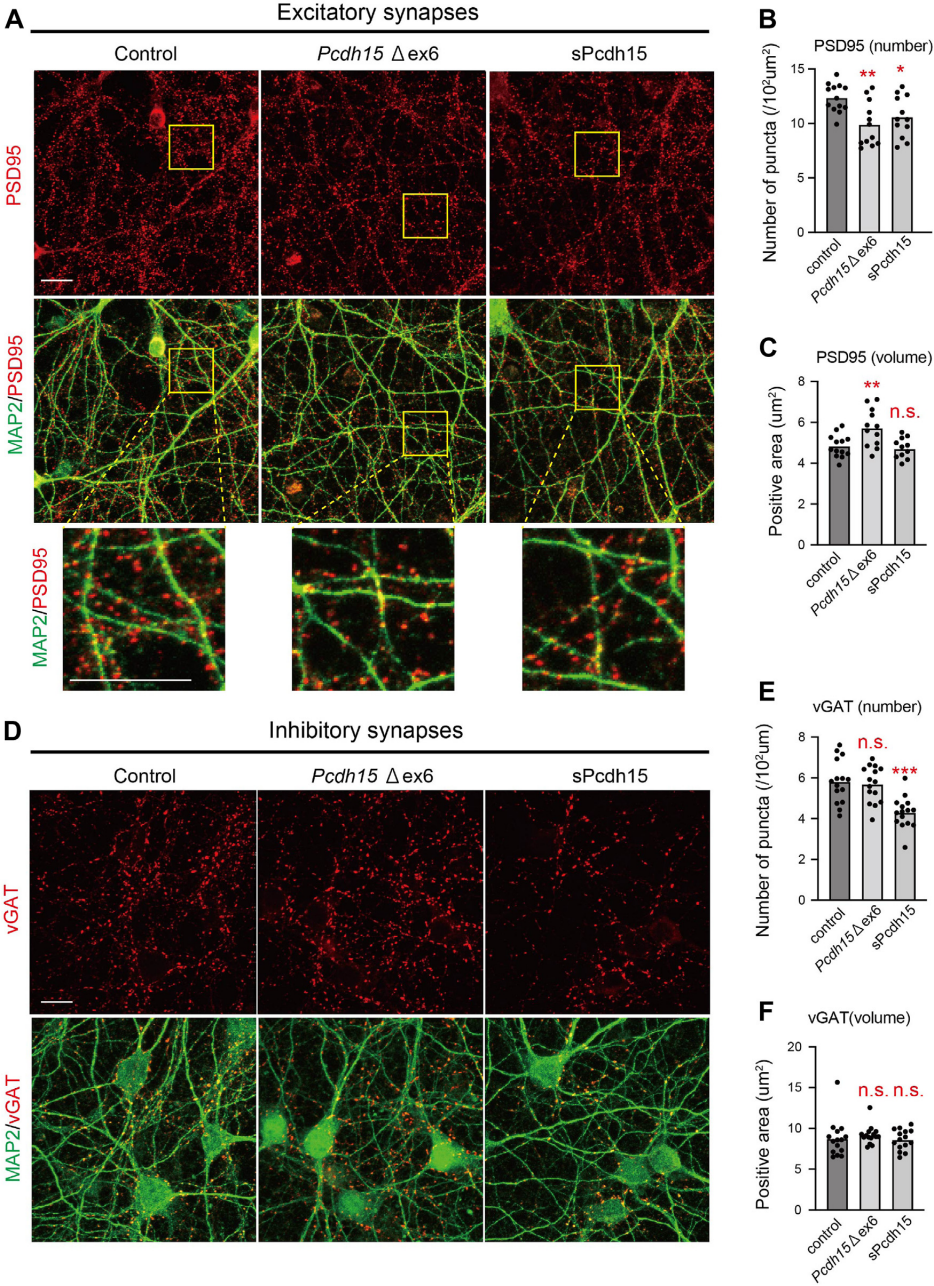
**The aberrant ALE isoform expression of *Pcdh15* influences excitatory and inhibitory synapses while the *Pcdh15* Δex6 mutation preferentially affects excitatory synapses.***A*, immunostaining of Cas9-expressing cortical neuronal cultures transduced with mock lentiviral vectors (control, *left*), lentiviral vectors carrying gRNAs to delete *Pcdh15* (*Pcdh15 Δex6*, *middle*), and *sPcdh15*-expressing vectors (sPcdh15, right) with the neuronal markers PSD95 and MAP2. The *upper panels* show PSD95-expressing puncta. The *middle panels* show an overlay between PSD95 and MAP2. The *lower panel* shows high-magnification images of the area surrounded by *dashed rectangles* in the *middle panels*. *B* and *C*, quantification of density (*B*) and volume (*C*) of excitatory synapses. n = 13, 13, and 12 fields for the control, *Pcdh15Δex6*, and sPcdh15, respectively. One-way ANOVA was followed by Tukey’s multiple comparisons test. *B*, synaptic number. *C*, synaptic volume. (*D*) immunostaining of Cas9-expressing cortical neuronal cultures transduced with mock lentiviral vectors (control, *left*), lentiviral vectors carrying gRNAs to delete *Pcdh15* (*Pcdh15Δex6*, *middle*), and *sPcdh15*-expressing vectors (sPcdh15, *right*) with the neuronal markers VGAT and MAP2. The *upper panels* show VGAT-expressing puncta. The *middle panels* show an overlay between VGAT and MAP2. *E* and *F*, quantification of density (*E*) and volume (*F*) of inhibitory synapses. n = 15 fields per group. One-way ANOVA was followed by Tukey’s multiple comparisons test. *E*, synaptic number. *F*, synaptic volume. The scale bars represent 10 μm in (*A* and *D*). ALE, ALE, alternative last exon; gRNA, guide RNA; pcdh15, protocadherin-15; PSD95,postsynaptic density 95; VGAT, vesicular gamma-aminobutyric acid transporter.

We next investigated the impact of aberrant *Pcdh15* ALE splicing on synapse development and compared it with *Pcdh15* mutation with a loss-of-function effect. Using the genome editing technique, we generated *Pcdh15* mutations by removing the constitutive exon 6, resulting in a frameshift truncation mutation (Fig. S3*A*). This mutation (Δex6) simulated a rare variant found in Usher syndrome patients [50-bp del NM_001384140.1(PCDH15): c.475–2204_594 + 1766 del] (19). The qPCR analysis confirmed the successful excision of *Pcdh*15 exon 6 in *Pcdh*15 Δex6 neurons and the overexpression of sPCDH15 in cortical neuron cultures led to a significant upregulation of *Pcdh15* (Fig. S3*B*). First, we examined the effects of the Δex6 mutation and ALE splicing alterations of *Pcdh15* on excitatory synapses. Immunostaining with anti-postsynaptic density 95 and anti-VGluT1 antibodies, which are excitatory postsynaptic markers, revealed a significant reduction in the number of synapses and an increase in spine size in the Δex6 mutant cultures (Figs. 5, *A*–*C* and S3*C*). The expression of sPCDH15 also led to a significant decrease in synapse numbers, resembling the phenotype of *Pcdh*15 Δex6 mutation but did not significantly affect spine size (Figs. 5, *A*–*C* and S3*C*). Additionally, there were no significant differences in the densities of the neurons [(microtuble associated protein 2 [MAP2]/4′,6-diamidino-2-phenylindole) double stained cells] (Fig. S3*D*) or transcript levels of synaptic markers (Fig. S3*E*) among groups, indicating that the altered number of excitatory synapses was not due to a reduced number of neurons or transcriptional changes.

Next, we analyzed the influences of the Δex6 mutation and ALE splicing alterations in *Pcdh15* on inhibitory synapses. Immunostaining with an anti-vesicular gamma-aminobutyric acid (GABA) transporter (VGAT) antibody, an inhibitory postsynaptic marker, showed that the expression of sPCDH15 significantly reduced the number of inhibitory synapses, while no significant changes were observed in the Δex6 mutant (Fig. 5, *D* and *E*). The spine size remained unchanged in both groups (Fig. 5*F*). Therefore, while the Δex6 mutation of *Pcdh15* selectively affects the excitatory synapses, the expression of sPCDH15 likely influenced both the excitatory and inhibitory synapses.

### Preferentially impact of aberrant ALE isoforms of *Pcdh15* on inhibitory synapses

**Figure 6:**
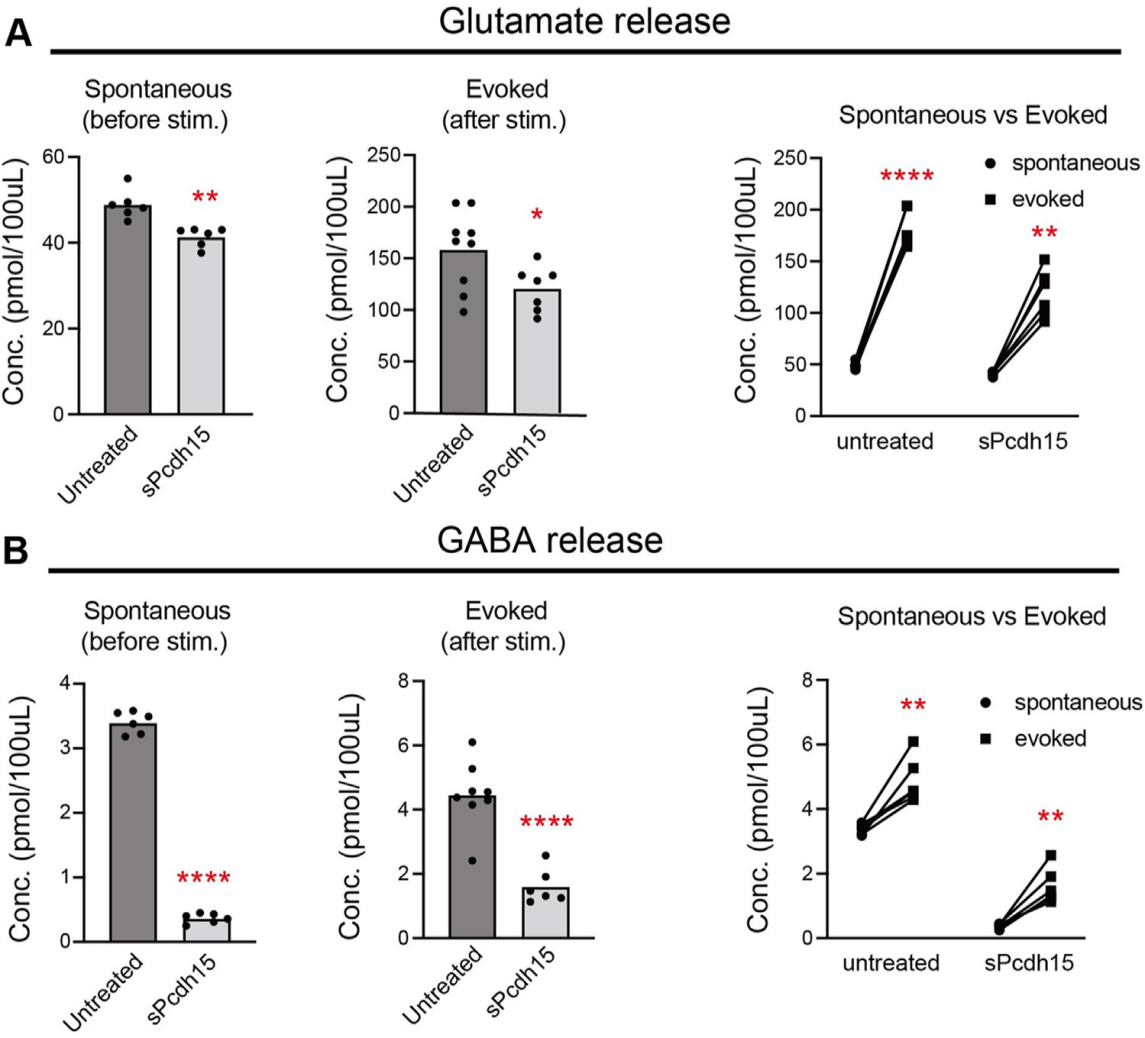
**The expression of sPCDH15 preferentially impairs inhibitory synapses**. *A* and *B*, concentrations of released glutamate (*A*) and GABA (*B*) in cortical neurons transduced with lentivirus-expressing mock genes (control) or *sPcdh15* (sPcdh15). The neurotransmitter level was measured upon a steady-stead level (spontaneous, *left panel*) and after the neurons had been depolarized with 4-AP (evoked, *middle panel*). The right panels show paired comparisons before and after neuronal stimulation in both groups. n = 8 and 6 cultures (control and sPcdh15), unpaired *t* test. The functional response to stimulation was checked using a paired comparison before and after stimulation (*right panels*). n = 6 cultures per group, paired *t* test. GABA, gamma-aminobutyric acid; NL2, neuroligin 2; sPCDH15, soluble form of PCDH15.

To confirm the effects of sPCDH15 on neuronal synapses, we measured excitatory and inhibitory neurotransmitters released in the expression cultures. LC-MS was used to quantify the spontaneous and evoked releases of neurotransmitters before and after pharmacological stimulations with 4-AP, respectively. While the spontaneous and evoked releases of glutamate showed a slight reduction in the sPCDH15-expressing cultures (Fig. 6*A*), the release of GABA exhibited a dramatic decrease (Fig. 6*B*). Furthermore, the releases of both neurotransmitters were significantly enhanced in the evoked cultures compared to the unstimulated cultures (Fig. 6, *A* and *B*), thereby confirming the functional synaptic response to depolarized stimulation. These data suggest that sPCDH15 exerts a more pronounced effect on inhibitory synapses compared to excitatory ones.

**Figure 7:**
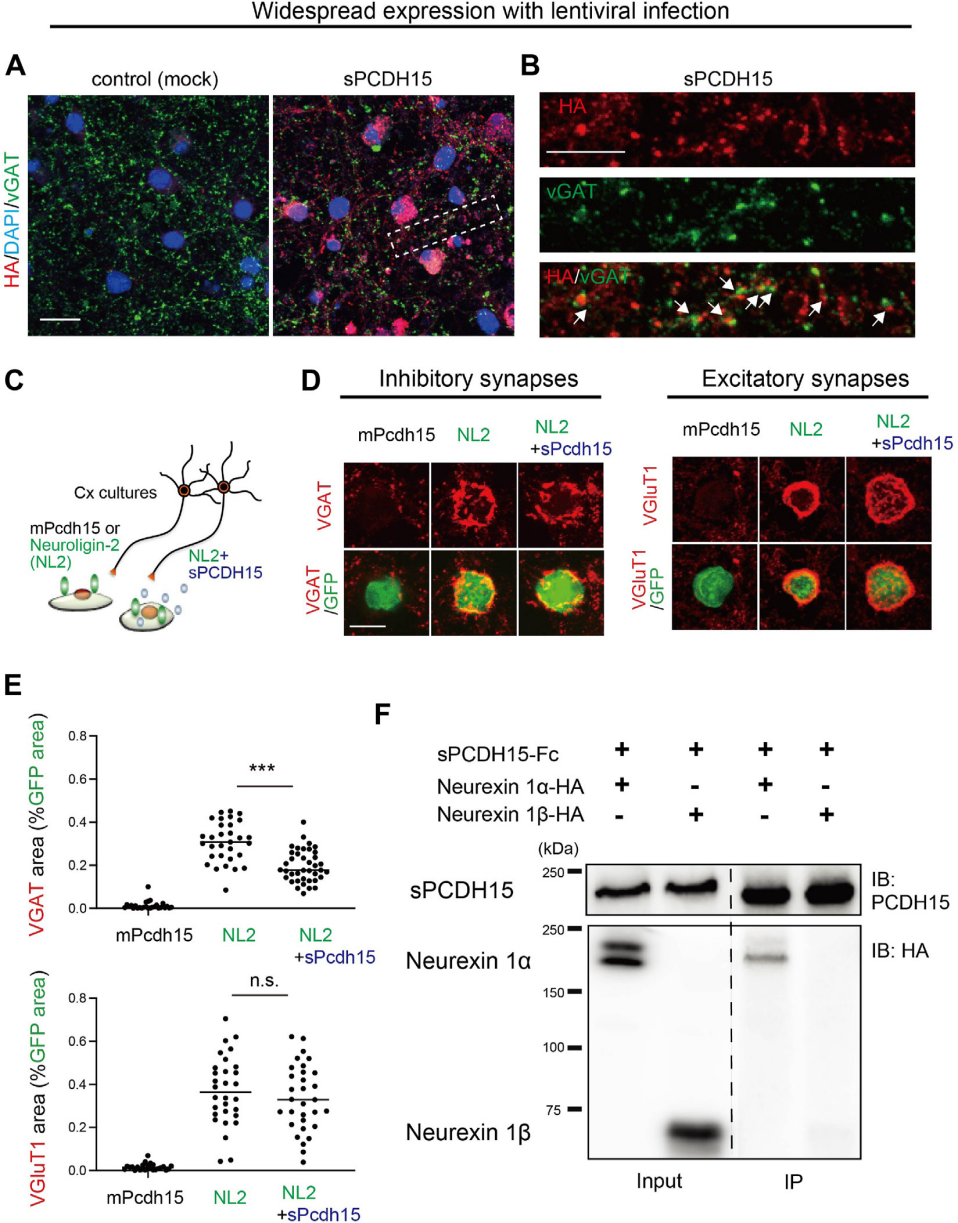
**Selective effects of the aberrant ALE isoform of PCDH15 on the formation of NL2-induced inhibitory synapses**. *A*, immunostaining of the subcellular localization of sPCDH15 in cultured cortical neurons transduced with lentiviral vectors expressing HA-tagged sPCDH15. VGAT and DAPI stained inhibitory synaptic boutons and neuronal nuclei, respectively. sPCDH15 was partially detected in the boutons of VGAT-positive inhibitory synapses. *B*, high magnification images of the area surrounded by *dashed rectangles* in (*A*). The *upper*, *middle*, and *lower* panels show the staining of sPCDH15 alone, VGAT alone, and overlay, respectively. *C*, cartoon showing the artificial synapse formation assay. NL2-HA was expressed into HEK293T cells with and without sPcdh15-HA and cocultured with cortical neurons. *D*, immunostaining of HEK293T cells expressing Pcdh15, NL2, and NL2+Pcdh15 with the presynaptic markers VGAT (inhibitory synapses, *left panel*) and VGluT1 (excitatory synapses, *right panel*). The overall morphology of cocultured HEK293T cells was visualized with a GFP. *E*, the quantification of synapse assembly described in (*D*) was achieved by measuring the VGAT or VGLUT1 areas relative to the GFP area. VGAT: [n = 27, 31, and 39 cells for mPcdh15, NL2, and NL2+sPcdh15, respectively]. VGluT1: [n = 25, 30, and 31 cells for mPcdh15, NL2, and NL2+sPcdh15, respectively]. One-way ANOVA was followed by Tukey’s multiple comparisons test. *F*, Western blots showing coimmunoprecipitation of neurexins-HA with sPCDH15-FC. Cells were transfected with sPCDH15-FC and either neurexin 1α or neurexin1β constructs as indicated at the top. Immunoprecipitation was performed with PCDH15-FC and bands were visualized by Western blotting using antibodies to HA and PCDH15. Protein extracts were analyzed for the expression of PCDH15 and neurexins without (*left*) and with (*right*) immunoprecipitation. The scale bars represent 10 μm in (*A*), 5 μm in (*B* and *D*). ALE, ALE, alternative last exon; DAPI, 4′,6-diamidino-2-phenylindole; HA, hemagglutinin; NL2, neuroligin 2; pcdh15, protocadherin-15; sPCDH15, soluble form of PCDH15; VGluT1, vesicular glutamate transporter 1; VGAT, vesicular gamma-aminobutyric acid transporter.

We further investigated the preferential effect of sPCDH15 on inhibitory synapses. Upon transient expression through *in vitro* electroporation, sPCDH15 was observed to be localized within the soma rather than synaptic boutons (Fig. 4, *A* and *B*). However, when sPCDH15 was widely expressed for a prolonged period with lentiviral infection [day *in vitro* (DIV) 7–14 for 7 days], immunoreactivity was partially colocalized with the anti-VGAT antibody, indicating some colocalization with inhibitory synapses in addition to its presence in the soma (Fig. 7, *A* and *B*). This implies that the aberrant ALE selection of sPCDH15 leads to the mislocalization of PCDH15 into inhibitory synapses.

To validate the preferential impact of sPCDH15 on inhibitory synapses, we investigated the effect of sPCDH15 on the synaptogenic activity induced by neuroligin 2 (NL2) *in vitro* (Fig. 7*C*). NL2 is a postsynaptic cell adhesion molecule known to preferentially organize inhibitory synapses (26, 27). However, when overexpressed in nonneuronal cells, NL2 can promote the formation of presynaptic contacts for both excitatory and inhibitory neurons in the artificial synapse formation assay (26). In our study, we observed that the expression of NL2 in HEK293T cells significantly assembled presynaptic contacts positive for both VGluT1 and VGAT. In contrast, the expression of mPCDH15 did not induce any synapse formation (Fig. 7, *D* and *E*). However, when sPCDH15 was coexpressed together with NL2, the assembly of VGAT, but not VGluT1, positive synapses were significantly attenuated (Fig. 7, *D* and *E*) indicating that sPCDH15 selectively prevents the formation of inhibitory synapses *in vitro*.

To gain insight into the mechanisms underlying the preferential inhibition of sPCDH15 on inhibitory synapses, we investigated its potential interaction with α-neurexins. Previous studies have demonstrated that α-neurexins exhibit a stronger association with NL2 and are predominantly localized to inhibitory synapses, compared to β-neurexins (28). Therefore, we hypothesized that sPCDH15 may preferentially interact with α-neurexins and hence disrupt α-neurexin-NL2 interaction that organizes inhibitory synapses. To address this hypothesis, we used a coimmunoprecipitation assay in which we transfected HEK293T cells with constructs encoding HA-tagged neurexin 1α or neurexin1β together with sPCDH15 encoding constructs. Following the pull-down of sPCDH15, we observed a specific coimmunoprecipitation with neurexin-1α, while no interaction was observed with neurexin-1β (Fig. 7*F*). The selective interaction between sPCDH15 and α-neurexins suggests a potential molecular mechanism underlying the observed preferential influence of sPCDH15 on inhibitory synapses. Together, these results provide insights into the neuronal impact of sPCDH15 and its preferential effect on inhibitory synapses.

## Discussion

### Differential mechanisms underlying SAM68-mediated ALE selection in neurons

The ALE isoforms of many genes are spatially and temporally altered in mammalian tissues. Recently, it was found that SAM68 is expressed in a tissue-specific manner and is required for the spatial control of ALE isoforms (10, 11, 12). Our study further demonstrated that SAM68 regulates ALE splicing of *Pcdh15* and *Il1rap* in a dose-dependent manner.

Recent studies have suggested that SAM68 synergistically regulates the 3′-end processing of target transcripts with U1 snRNP complexes (11, 13) by protecting pre-mRNAs from drastic premature termination by cleavage and PASs in introns (14, 22). Consistently, our data suggested that the ALE splicing of *Il1rap* was dependent on U1 snRNP in neurons (Fig. 2). This is conceivable because U1 motif-like sequences were located around the cryptic PASs of *Il1rap.* In contrast to *Il1rap*, the ALE splicing of *Pcdh15* was, however, resistant to U1 AMO and unlikely dependent on U1 snRNP because no U1 motif-like sequence was found around the cryptic PASs of *Pcdh15*.

Our data suggest that SAM68-mediated ALE splicing of target transcripts is not only regulated by SAM68-U1 snRNP interaction but also can be modulated by external regulations in neurons. Consistent with our previous findings that SAM68 regulates neuronal activity–dependent alternative splicing through the CaMK pathway (23), we found that SAM68-regulated ALE selection is affected similarly. We found that the ALE selection of *Pcdh15*, but not *Il1rap*, was partially affected by the depletion of spontaneous activity and blockade of CaMK-dependent phosphorylation of SAM68 (Fig. 3). Thus, SAM68-regulated ALE splicing appears to be complex and likely contingent upon the specific target transcript being involved.

It is conceivable that the differential regulation of ALE splicing by SAM68 among target transcripts is likely determined by the presence or absence of neighboring cofactors such as U1 snRNP. Besides, we speculate that the cooperative action of U1 snRNP and SAM68 confers ALE regulation greater stability and reduced susceptibility to external regulation, whereas the sole regulation by SAM68 is likely to be more prone to external effects.

### Impact of SAM68-dependent ALE splicing of *Pcdh15* on synaptic function

The role of PCDH15 in the central nervous system including its expression, localization, and functions remains poorly understood. However, mutations, copy number variations , and single nucleotide polymorphisms in PCDH15 have been associated with a range of neurological disorders such as autism, bipolar disorder, schizophrenia, and Usher syndrome (17, 18, 20, 21, 29). In our study, we reported that PCDH15 is highly localized in excitatory postsynapses, and a pathogenic loss-of-function mutation caused a reduction in the excitatory synapses (Figs. 4 and 5). This finding is consistent with a recent study that demonstrated a decrease in the number of synapses in human induced pluripotent stem cell–derived neurons from two bipolar disorder patients with a small deletion in the *PCDH15* gene (30). This suggests that PCDH15 may play a pivotal role in various aspects of synaptic functions, and the misspliced *Pcdh15* may contribute to the pathologies of several psychiatric disorders. Interestingly, we found that the expression of the aberrant ALE isoform, sPCDH15, slightly disrupted the formation and function of excitatory synapses, resembling the pathogenic loss-of-function mutation.

Compared to its influence on excitatory synapses, sPCDH15 preferentially affected inhibitory synapses (Figs. 5 and 6). In addition, sPCDH15 significantly prevented NL2-induced inhibitory synapse formation *in vitro* (Fig. 7, *D* and *E*) and dramatically reduced the spontaneous and evoked releases of GABA. The preferential effects of sPCDH15 on inhibitory synapses may be attributed to its mislocalization around inhibitory synapses in contrast to mPCDH15 which is localized around excitatory synapses. Furthermore, the physical interaction between sPCDH15 and α-neurexins, which are predominantly localized to inhibitory synapses and form trans-synaptic association with NL2, could contribute to this preferential effect. This aligns with recent studies demonstrated that a clustered PCDH, γ-Pcdh, negatively regulates inhibitory synapse density *in vivo* (31, 32) by disrupting the interaction between NLs and neurexins. Importantly, we found that the loss-of-function mutation of PCDH15 did not affect inhibitory synapses (Fig. 5, *D* and *E*). Therefore, we speculate that the impact of sPCDH15 on inhibitory synapses may represent a gain-of-function effect mediated through its physical interaction with neurexin 1α. Given that neurexin 1α is implicated in several neurodevelopmental disorders such as autism spectrum disorder and schizophrenia (33, 34), the aberrant interaction between PCDH15 isoforms and neurexin 1α might serve as a potential pathological mechanism underlying the impact of PCDH15 mutations associated with neurological diseases on neuronal functions. Further investigations are warranted to explore the interaction between PCDH15 mutations and variants reported in neurological diseases and synaptic organizers such as neurexins to test this hypothesis.

Given that altered 3′UTR exons in *Sam68* KO brains include a considerable number of transcripts encoding transmembrane or secreted proteins other than PCDH15 and IL1RAP with neuronal functions, it is conceivable that ALE selection by SAM68 may be involved in a plethora of neuronal and synaptic functions.

## Experimental procedures

### Antibodies and plasmids

The following commercially available antibodies were used for the immunostaining analyses: mouse anti-HA (clone HA-7, Sigma-Aldrich), rat anti-HA (clone 3F10, Sigma-Aldrich), mouse anti-PSD95 (clone K28/43, NeuroMab), rabbit anti-MAP2 (Sigma-Aldrich), rabbit anti-VGluT1 (#1353303, Synaptic Systems), and guinea pig anti-VGAT (#676780, Calbiochem) and rabbit anti-PCDH15 (16). Secondary antibodies with minimal interspecies cross-reactivity conjugated to Alexa Fluor dyes (Molecular Probes) were used for visualization in the immunostaining analyses or conjugated with horseradish peroxidase (Cell Signaling Technology) for Western blotting. To visualize the overall morphology of transfected HEK293T cells in artificial synapse formation assay (see below), we used previously described expression vectors comprising GFP, NL2 (containing the A site), mPcdh15, and sPcdh15 (pMAX-GFP, pCAGGS-NL2A-HA, pCAGGS-mPcdh15-HA, and pCAGGS-sPcdh15-HA) (10, 35).

To construct *Il1rap* minigene, the DNA fragment of *Il1rap* exon 1-intron 8 was amplified by PCR from NIH3T3 genome using IL1RAP E x 1 for Bam (5′-GAGTCTGTCCTTCTATGGGATC-3′) and IL1RAP int8 rev RI (5′-ggcggcGAATTCcctgtacttgagcaatctggat-3′) primers, and the DNA fragment of Il1rap intron 12-exon 13 was amplified by PCR from NIH3T3 genome using IL1RAP int12 for RI (5′-ggcggcGAATTCgaggaactgggtaacagcagc-3′) and IL1RAP E x 13 rev XhoI (5′-GGCGGCCTCGAGctcccatgaaccgccttgttc-3′) primers. The DNA fragment of Il1rap exon 1-intron 8 and the DNA fragment of Il1rap intron 12-exon 13 were digested with BamHI and EcoRI, and EcoRI and XhoI, respectively, and subcloned into pcDNA3.1-Myc-HIS (Thermo Fischer Scientific). To construct *Pcdh15* minigene, the DNA fragment of Pcdh15 exon 26-intron 26 was amplified by PCR from NIH3T3 genome using *pcdh15* E x 25 for Kpn (5′- GGCGGCGGTACCgctggattccagACAAGTTTGG-3′) and *pcdh15* Int25 rev Bm (5′- GGCGGCGGATCCacagcctttcaaagggagatgc-3′) primers, the DNA fragment of Pcdh15 intron 26-exon 26-intron 26 was amplified by PCR from NIH3T3 genome using pcdh15 Int25 for Bm (5′- GGCGGCGGATCCagaaaaatgggggacattctatgc-3′) and pcdh15 Int26 rev Xho (5′-GCCGCCCTCGAGcccaaccttaatcctaggactg-3′) primers, and the DNA fragment of *Pcdh15* intron 26-exon 27 was amplified by PCR from NIH3T3 genome using pcdh15 Int26 for Xho (5′-GGCGGCCTCGAGcaactcaaaaggtggcagttgg-3′) and *pcdh15* E x 27 rev Xba (5′- GGCGGCTCTAGActtcactctgagcacagatgcg-3′) primers. the DNA fragment of *Pcdh15* exon 26-intron 26, the DNA fragment of *Pcdh15* intron 26-exon 26-intron 26, and the DNA fragment of *Pcdh15* intron 26-exon 27 were digested with KpnI and BamHI, BamHI and XhoI, and XhoI and XbaI, respectively, and subcloned into pcDNA3.1-Myc-HIS (Thermo Fischer Scientific).

### RNA isolation, semi-qPCR, and RT-qPCR assays

For the qPCR analyses, RNA was harvested from cultured neurons using Nucleospin RNA XS (Takara) or RNAiso Plus reagent (Takara), followed by the removal of contaminating DNA using Turbo DNA-free (RNase-free DNase; Ambion). Total RNA (1–2 μg) was reverse transcribed using random hexamers and the PrimeScript first strand cDNA Synthesis Kit (Takara). Reverse transcription-quantitative PCR (RT-qPCR) was performed using a StepOnePlus qPCR system (Applied Biosystems) with Power SYBR Green PCR Master Mix (Applied Biosystems) and the comparative CT method. For relative quantification using qRT-PCR, transcript levels were normalized to that of *Gapdh* and relative quantification values were calculated. The oligonucleotide primers used for the qPCR are listed in Table S1.

For the abundance ratio of the SF to LF, the percentage of the SF variant was largely estimated from the CT value (CT^SF^) and directly compared to that of the LF (CT^LF^) at the same threshold set for the CT value. The CT^LF^ + CT^SF^ values for the total transcript levels were set to 100%.

### ALE mini-gene reporter assay

The expression vectors for the *Pcdh15* and *Il1rap* ALE reporters were transfected with WT or phospho-mutant SAM68 plasmids using X-tremeGENE DNA Transfection Reagent (Roche Applied Science) into HEK293T cells. To measure the effect of the expression of SAM68 and its variant on ALE reporter splicing, RNA isolation and RT-qPCR assays were performed as described above.

### Western blot and immunoprecipitation

HEK293T cells were transfected with the expression vectors using X-tremeGENE DNA Transfection Reagent (Roche Applied Science) following the manufacturer’s instructions. After 24 h, the transfected cells were lysed with RIPA buffer (25 mM Tris–HCl, pH 8.0, 150 mM NaCl, 1% NP-40, 1% deoxycholate, 0.1% SDS) supplemented with a protease inhibitor cocktail (Roche Applied Science). The resulting lysates were centrifuged at 4 °C, and the supernatants were collected. The supernatants were then mixed with sample buffer, subjected to SDS-PAGE electrophoresis, and transferred into polyvinylidene fluoride membranes for immunoblotting. The membranes were probed with the corresponding primary antibodies, followed by incubation with horseradish peroxidase-conjugated secondary antibodies. The protein signals were visualized with the enhanced chemiluminescence detection kits (Pierce) and captured with an image analyzer (LAS500; GE HealthCare). For immunoprecipitation, the collected supernatants were incubated overnight with the indicated antibodies, and then protein G-Sepharose beads (Sigma-Aldrich) were added for 1 h following the manufacturer’s instructions. The beads were subsequently washed three times with PBS and boiled in 1 × sample buffer before Western blot analysis.

### Neuronal cell culture and artificial synapse formation assays

Cortical neuron cultures were prepared as previously described (36). The cultures were prepared from Institute of Cancer Research or Cas9-expressing mouse pups on embryonic day 15. The tissues were dissociated with 0.05% trypsin (Sigma-Aldrich) in the presence of DNase I (Roche Applied Science) for 10 min at 37 °C. After the cells had been dissociated, trypsin was inactivated with a soybean trypsin inhibitor (Sigma-Aldrich). The cells were then plated into poly-D-ornithine-coated dishes (2.0 × 10^5^/cm^2^) and maintained for 15 days in Neurobasal medium (Invitrogen) containing 2% B27 supplement, 2 mM of glutamax, and penicillin/streptomycin (Invitrogen). For the pharmacological experiments, TTX, bicuculine , STO609, KN93, U0126, and PP2 purchased from TOCRIS or Sigma-Aldrich were added one day before harvesting. All procedures related to the care and treatment of animals were carried out in strict accordance with the Guide for the Care and Use of Laboratory Animals of Tokai University. All mice were maintained under specific pathogen-free conditions at the Laboratory Animal Center, Tokai University. The experimental protocol was approved by the Institutional Animal Care and Use Committee of Tokai University (permit number 224036). All surgeries were performed under sodium pentobarbital anesthesia with efforts made to minimize animal suffering.

For the immunostaining analyses, cultured neurons were fixed with 4% paraformaldehyde in ice-cold PBS for 15 min. The neurons were then permeabilized with PBS containing 0.15% TritonX-100 for 15 min at room temperature and incubated with blocking solution (5% normal goat serum in PBS) for at least 30 min at room temperature. They were then incubated with the primary antibodies for 24 h at 4 °C. The appropriate secondary antibodies conjugated to Alexa 546 or 488 (goat; 1:1000) (Life Technologies) were used for visualization.

For the artificial synapse formation assay, HEK293T cells coexpressing NL2 with GFP were plated on the cortical neuron cultures at DIV14 (1–2 x 10^4^ cells/cm^2^) and immunostained with the presynaptic markers and anti-VGluT1 and anti-VGAT antibodies.

### Image acquisition and analysis

Confocal images of the neuronal cell cultures were captured using an LSM700 confocal system (Zeiss). The original images were analyzed using ImageJ (NIH) (https://imagej.nih.gov/ij/index.html). The number and size (area) of the synapse marker-positive puncta defined with proper thresholding (top 2.5–5.0% of the display range) were quantified. The morphologies of the neurons were visualized by staining with the neuronal marker MAP2. Nuclei were visualized with 4′,6-diamidino-2-phenylindole stains.

Quantification in the artificial synapse formation assay was performed as previously described (35, 36). In brief, after the appropriate threshold had been set, the immunoreactive areas on the surface of the HEK293T cells were measured. Approximately 20 to 40 cells from more than 10 separate fields per culture were quantified per group. The morphologies of the HEK293T cells were visualized by coexpressing GFP.

### Lentiviral production and generation of vectors for *in vitro* genome editing

The procedures for lentiviral production have been previously described (37). pCL20c vectors were designed under the control of the murine stem cell virus promoter (38). The viral vector was produced by cotransfecting HEK293T cells with a mixture of four plasmids using a calcium phosphate precipitation method. The four-plasmid mixture consisted of 6 μg of pCAG-kGP1R, 2 μg of pCAG-4RTR2, 2 μg of pCAG-VSV-G, and 10 μg of the vector plasmid pCL20c (pCL20c-MSCV-sPcdh15-HA-IRES-EGFP). The medium containing vector particles was harvested 40 h after transfection. Media samples were concentrated by centrifugation at 15,000*g* for 5 to 7 h. Viral samples were then suspended in Neurobasal medium, frozen in aliquots, and stored at −80 °C until further use. After assessing the titer in the HEK293T cells, the appropriate amount of lentivirus was transduced into cultured neurons 5 to 7 days before harvesting the cells.

For the design of the lentiviral vectors for the *in vitro* genome editing, the sequences of guide RNAs were selected based on CHOPCHOP (https://chopchop.cbu.uib.no) (>Rank5) (see Table S1). The Lentiviral Clustered Regularly Interspaced Short Palindromic/CRISPR-associated proteins 9 System (System Biosciences LLC) was then used according to the manufacturer’s protocol to construct the vector, and the oligonucleotides of the guide RNAs were inserted into the U6 promoter-driven lentiviral vector (CASLV511PA-G).

### AMOs and electroporation

The sequences of U1 and the control AMOs were used as previously described (14). The AMOs were purchased from Gene Tools, LLC (Philomath). AMO transfection was performed using the Neon Transfection System (Life Technologies). The cells (2 × 10^7^ cells) were resuspended in 100 μl of the resuspension buffer containing the AMOs. They were then electroporated and plated onto 12- or 24-well culture dishes. Electroporation was performed using the following parameters based on manufactural protocol: 1500 V (pulse voltage), 10 msec (pulse width), and three pulses.

### Measurement of neurotransmitter release by LC-MS

The culture supernatant was collected, dried using a SpeedVac vacuum concentrator (Thermo Fischer Scientific), and reconstituted in 100 μl of methanol, of which 10 μl were injected into an LC-MS. A quantitative analysis of the neurotransmitters was performed using an LCMS-8050 triple quadrupole mass spectrometer coupled to the ultra-high-performance liquid chromatography-Nexera system (Shimadzu). The chromatographic condition of the neurotransmitters was optimized using modifications described in previously published procedures (39, 40). The samples were separated in a hydrophilic interaction liquid chromatography mode using a 2.1 × 150 mm, 3.5 μm iHILIC Fusion(P) zwitterionic column (HILICON AB). Mobile phase A consisted of 100 mM of ammonium bicarbonate containing 5 μM of medronic acid (pH 9.0). Mobile phase B included 90% LC–MS-grade acetonitrile containing 10 mM of ammonium bicarbonate and 5 μM of medronic acid (pH 9.0). Linear gradient elution was achieved using the following procedure: 0 to 2 min, 95% B; 2 to 10 min, 95 to 5% B; 10 to 12.5 min, 5% B. The gradient was then restored to the initial composition (95%) for equilibration before the next run. The total flow rate was 2.0 ml/min. Selected reaction monitoring in positive electrospray ionization mode was performed to detect GABA (m/z 104.15 > 87.10 for quantitation, m/z 104.15 > 69.10 for identification) and glutamic acid (m/z 148.15 > 84.10 for quantitation, m/z 148.15 > 56.10 for identification).

### Statistical analysis

GraphPad Prism 5 (GraphPad Software) (http://www.graphpad.com/scientific-software/prism/) was used for the statistical analyses. D'Agostino-Pearson or Shapiro–Wilk normality tests were used to ensure the data had been normally distributed. Comparisons between two groups were made using Student’s *t*-tests, and those between three or more groups were made using an analysis of variance (ANOVA) followed by Tukey’s or Dunnett’s tests for pairwise comparisons unless otherwise stated. Data were presented as individual data points and means. Significance levels were indicated as follows: ∗∗∗∗*p* < 0.0001; ∗∗∗*p* < 0.001; ∗∗*p* < 0.01; ∗*p* < 0.05. Statistical significance between each experimental group and the control group (untreated/mock/WT) was indicated above the column of the respective experimental group and significance between experimental groups was represented by lines flanking the two groups being compared.

## Data availability

The data that support the findings of this study are available from the corresponding author (T. I.) upon reasonable request. The RNA-seq data can be accessible at the DDBJ database (6781), with the accession number of DRA6781, with the bioproject accession number of PRJDB6781.

## Supporting information

This article contains supporting information.

## Conflict of interest

The authors declare that they have no conflicts of interest with the contents of this article.

## Supporting information

Table S1

Supprting Information
