# Supplementary material for: Neuronal SAM68 differentially regulates alternative last exon splicing and ensures proper synapse development and function
Source: J Biol Chem. 2023 Aug 16;299(10):105168. doi: 10.1016/j.jbc.2023.105168 (PMC10562862; doi:10.1016/j.jbc.2023.105168)
Supplement: Table S1 [file mmc1.docx]

**RNA oligonucleotide sequences for guide RNAs**

| Forward primer  Reverse primer | Sequence |
| --- | --- |
| *Pcdh15 gRNA1 S*  *Pcdh15 gRNA1 AS* | 5'- ATC CGA TCC GGT AAG TAC TCA GGG -3'  5'- AAA CCC CTG AGT ACT TAC CGG ATC -3' |
| *Pcdh15 gRNA2 S*  *Pcdh15 gRNA2 AS* | 5'- ACC GCG AGA ACC CCG TGA ATA TCG -3'  5'- AAA CCG ATA TTC ACG GGG TTC TCG -3' |

**Oligonucleotide sequences of primer sets for semi-quantitative PCR**

| Forward primer  Reverse primer | Sequence | products (bp) |
| --- | --- | --- |
| *Pcdh15 ex5*-F  *Pcdh15 ex7*-R | 5'- TGT TGG GAC AGA TGA CAT CGC C -3'  5'- GAG AGC TGG CCC TGG AAG GG -3' | 250/158 |
| *Gapdh*-F  *Gapdh*-R | 5'- TGT TGC CAT CAA TGA CC -3'  5'- TCT CAT GGT TCA CAC CCA -3' | 342 |

**Oligonucleotide sequences of primer sets for RT-qPCR**

| Forward primer  Reverse primer | Sequence | products (bp) |
| --- | --- | --- |
| *Pcdh15* ex25-F  *Pcdh15* ex26-R  *Pcdh15* ex27-R | 5’- CCG GGT ACA AGC AGA TTC TC -3’  5’- TTG ACA CCT GGG TTC TCC AT -3’  5’- GGG TGA TCG TTT TCA TCC TG -3’ | 108/101 |
| *Pcdh15* total  (ex25)-F  *Pcdh15* total  (ex25)-R | 5’- TGG ATT ACG AGA CAA GGA CCA -3’  5’- TTG AAG GGA CTC GGA GAT TG -3’ | 87 |
| *Il1rap* ex8-F  *Il1rap* ex9-R  *Il1rap* ex8b-R  *Il1rap* ex13-R | 5’- GCT GCC AAG GTG AAA CAG A -3’  5’- GGA CCA TCT CCA GCC AGT AA -3’  5’- GTG TTT TGT GTC CGA TGT GG -3’  5’- TGG AGC ACG TAG TTG GGA CT -3’ | 130/127/101 |
| *Il1rap* total (ex3)-F  *Il1rap* total (ex3)-R | 5’- ACT ACA GCA CTG CCC ATT CC -3’  5’- CGG AAC CAG AGC ACA TCT TT -3’ | 136 |
| *Sam68*-F  *Sam68*-R | 5’- AAG AAC GCG TGC TGA TAC CT -3’  5’- GCA CCA GTC TCT TCC TGG AG -3’ | 109 |
| *Pcdh15*-6F  *Pcdh15*-7R2 | 5’- CAC GAT ATT CAC GGG GTT CT -3’  5’- GTC GTT GGA TGT CGG ATC TT -3’ | 115 |
