# Supplementary material for: Neuronal SAM68 differentially regulates alternative last exon splicing and ensures proper synapse development and function
Source: J Biol Chem. 2023 Aug 16;299(10):105168. doi: 10.1016/j.jbc.2023.105168 (PMC10562862; doi:10.1016/j.jbc.2023.105168)
Supplement: Supprting Information [file mmc2.docx]

**Supporting Information**

**Neuronal SAM68 differentially regulates alternative last exon splicing and ensures proper synapse development and function**

Mohamed Darwish^1,2^, Masatoshi Ito^3^, Yoko Iijima^1,4^, Akinori Takase^3^, Noriko Ayukawa^4^, Satoko Suzuki^4^, Masami Tanaka^4^, Kanae Komori^5^, Daisuke Kaida^5^ and Takatoshi Iijima^1,4,*^

^1^ Department of Molecular Life Science, Division of Basic Medical Science and Molecular Medicine, School of Medicine, Tokai University, Kanagawa 259-1193, Japan

^2^ Department of Biochemistry, Faculty of Pharmacy, Cairo University, Cairo, 11562, Egypt

^3^ The Support Center for Medical Research and Education, Tokai University,

Kanagawa 259-1193, Japan

^4^ Tokai University Institute of Innovative Science and Technology, Isehara, Kanagawa, Japan

^5^ Graduate School of Medicine and Pharmaceutical Sciences, University of Toyama, Toyama 930-0194, Japan

*Corresponding author: Takatoshi Iijima, Ph.D

School of Medicine, Tokai University

143 Shimokasuya, Isehara City, Kanagawa 259-1193, Japan

Tel.: +81-463-93-1121; E-mail: takatoshi.iijima@tokai-u.jp

**Supplementary Figures and Tables**

**Figure S1: Neuronal SAM68-regulated ALE selections**

Additional information related to Figure 1.

**Figure S2: U1 snRNP distinctly regulates SAM68-regulated ALE selections**

Additional information related to Figure 2.

**Figure S3: Generation and characterization of *Pcdh15* Δex6 mutants using the CRISPR/Cas9 system**

Additional information related to Figure 5

**Supplementary Tables**

**Table S1. List of PCR primer sets**

**Supplementary Figures and Tables**

**Figure S1: Neuronal SAM68-regulated ALE selections (Related to Figure 1)**

(A) Aberrant 3’UTR exon selection of the representative genes in *Sam68* ^KO^ brains. The upper panels show a schematic illustration of the selection of the ALE in *Cp* (exon 18) and *Lrrcc1* (exon 19). Lower panels show sashimi plots of the ALE events in the relevant genes. Each plot includes cassette exons and intron retentions. The red plots represent the wild-type mice, and the green plots represent *Sam68* ^KO^. The X-axes show genomic loci, and the Y-axes indicate transcription intensity. A “sashimi-like” region in each plot indicates an exonic region, and the blank regions between them indicate intronic regions. The numbers on the bridges crossing exons indicate junction reads. (B) Aberrant ALE selection of the ALE of *Il1rap*, *Pcdh15*, *Cp*, and *Lrrcc1* genes in Sam68^KO^ brains shown in exon array. Data of exon array based on the UCSC genome browser Mouse July 2007 (NCBI37/mm9) assembly.

**Figure S2: U1 snRNP distinctly regulates SAM68-regulated ALE selections (Related to Figure 2)**

(A) The full-length cDNA sequences of *Il1rap* exon 8 and *Pcdh15* exon 26. Green colors indicate the coding exon region. Blue colors show putative PASs on the 3’UTR. Yellow colors show putative U1 motifs. (B) Relative expression of the long-form (LF) variants of *Il1rap* and *Pcdh15* in cultured cortical neurons treated with different concentrations of U1 AMOs by RT-qPCR. The Ct value of each alternative isoform was normalized to the total transcripts. n = 3–6 experiments per condition. One-way ANOVA was followed by Tukey’s multiple comparisons test. (C) Relative expression of *Khdrbs1* (*Sam68*) between different concentrations of U1 AMOs by RT-qPCR. The Ct value of each alternative isoform was normalized to *Gapdh*. n = 4 experiments per condition. (D-F) Immunostaining of cortical neuronal cultures at DIV10 electroporated with U1 AMOs (0 µM and 20 µM) with the neuronal markers PSD95 and MAP2. (B) The upper panels show PSD95-expressing puncta. Middle panels show overlay between PSD95 and MAP2 (C) The number of neurons (MAP2/DAPI^+^ cells). (n = 10 fields per group) (D) The density of PSD95-positive excitatory synapses electroporated with U1 AMOs (0 µM and 20 µM). (n = 10 fields per group). A student’s t-test was used. Scale bars = 50 μm in (B).

**Figure S3: Generation and characterization of *Pcdh15* Δex6 mutants using the CRISPR/Cas9 system (Related to Figure 5)**

(A) Left, schematic illustration showing the design of deleting *Pcdh15* gene locus with CRISPR/Cas9 system. The upper part shows the location of the designed gRNAs and the subsequent effect on exon 6. Arrows show the design of primers used to confirm the deletions. The lower part shows the effect of exon 6 deletion on protein truncation. Right, semi-quantitative RT-PCR of *Pchd15* gene in neurons with *Pchd15* Δex6 mutation and the controls. (B) qPCR analysis of *Pcdh15* mRNA derived from control, *Pchd15* Δex6, *sPcdh15* overexpressin*g* neurons*.* *Gapdh* was used as an internal control. (C) The density of excitatory synapses from control, *Pchd15* Δex6, *sPcdh15* overexpressin*g* neurons. n = 8 fields per group. A one-way ANOVA was followed by Turkey’s multiple comparisons test. (D) The number of neurons (MAP2^+^/DAPI^+^ cells) counted from control, *Pchd15* Δex6, *sPcdh15* overexpressin*g* neuron cultures. n = 26 fields per group. One-way ANOVA was followed by Turkey’s multiple comparison test. (E) qPCR analysis of mRNA of major synaptic markers derived from control, *Pchd15* Δex6, *sPcdh15* overexpressin*g* neurons. *Gapdh* was used as an internal control. n = 4 cultures per group. One-way ANOVA was followed by Turkey’s multiple comparison test.
