# Supplementary material for: Neuronal SAM68 differentially regulates alternative last exon splicing and ensures proper synapse development and function
Source: J Biol Chem. 2023 Aug 16;299(10):105168. doi: 10.1016/j.jbc.2023.105168 (PMC10562862; doi:10.1016/j.jbc.2023.105168)
Supplement: Supplemental figures [file mmc3.pdf]

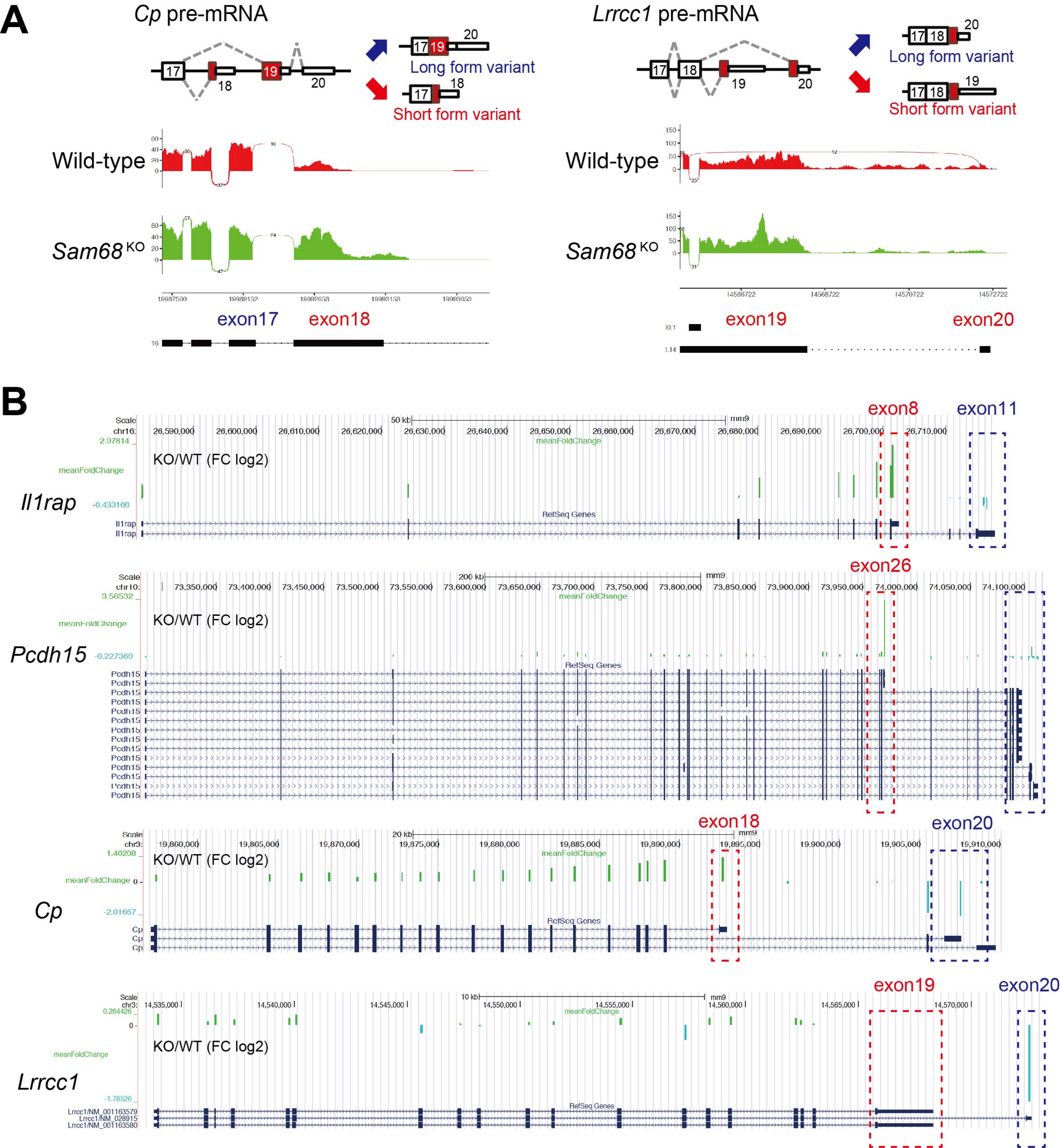

A
Cp pre-mRNA
Long form variant
Short form variant
Wild-type
Sam68 KO
exon17
exon18
Lrrcc1 pre-mRNA
Long form variant
Short form variant
Wild-type
Sam68 KO
exon19
exon20
B
exon8
exon11
KO/WT (FC log2)
Il1rap
exon26
KO/WT (FC log2)
Pcdh15
exon18
exon20
KO/WT (FC log2)
Cp
exon19
exon20
KO/WT (FC log2)
Lrrcc1
RefSeq Genes
meanFoldChange

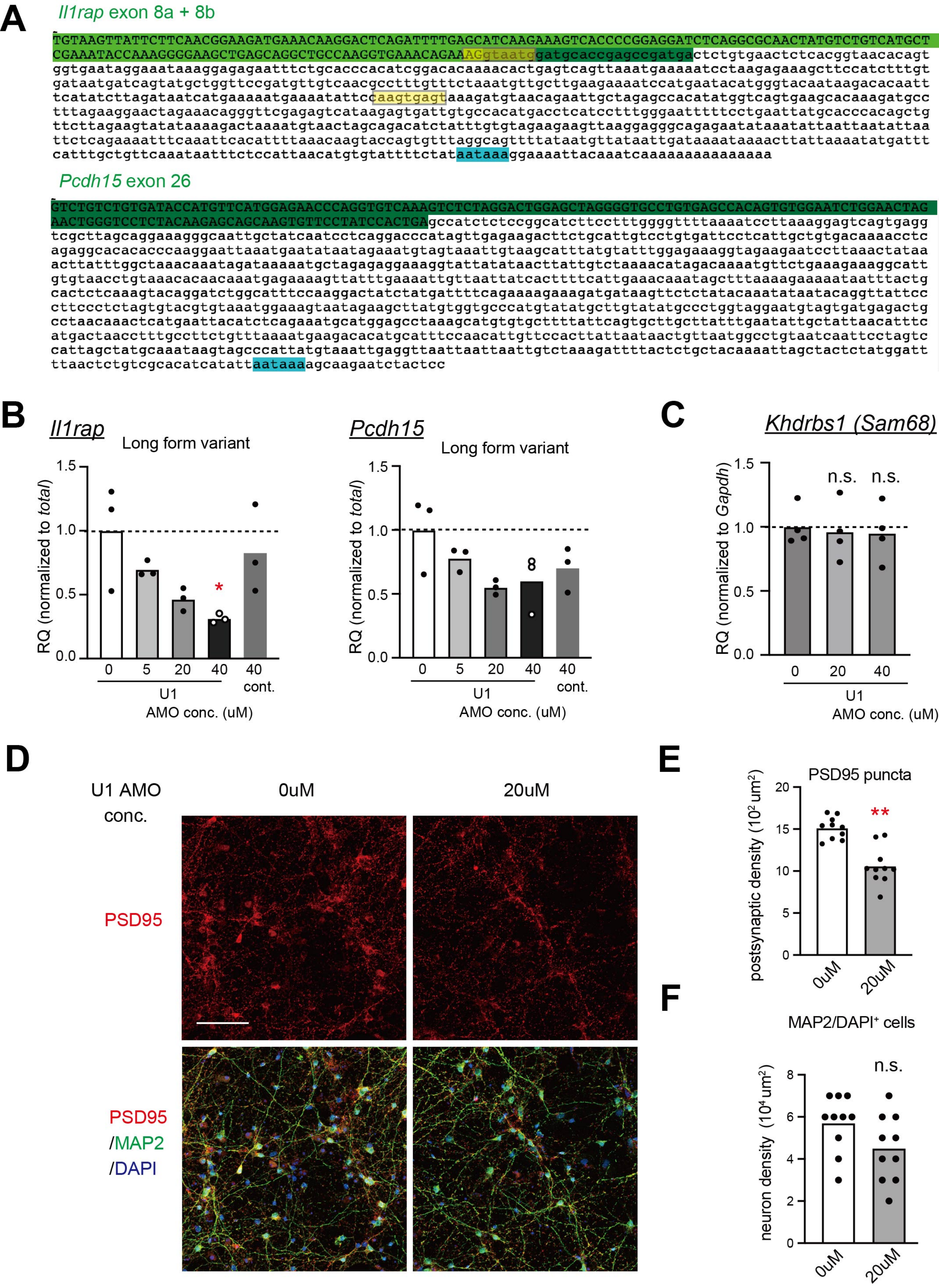
A
Il1rap exon 8a + 8b
TGTAAGTTATTCTTCAACGGAAGATGAAACAAGGACTCAGATTTTGAGCATCAAGAAAGTCACCCCGGAGGATCTCAGGCGCAACTATGTCTGTCATGCT
CGAAATACCAAAGGGGAAGCTGAGCAGGCTGCCAAGGTGAAACAGAAAGgtaatggatgcaccgagccgatgactctgtgaactctcacggtaacacagt
ggtgaataggaaataaaggagagaatttctgcacccacatcggacacaaaacactgagtcagttaaatgaaaaatcctaagagaaagcttccatctttgt
gataatgatcagtatgctggttccgatgttgtcaacgcctttgtttctaaatgttgcttgaagaaaatccatgaatacatgggtacaataagacacaatt
tcatatcttagataatcatgaaaaatgaaaatattccaagtgagtaaagatgtaacagaattgctagagccacatatggtcagtgaagcacaaagatgcc
tttagaaggaactagaaacagggttcgagagtcataagagtgattgtgccacatgacctcatcctttgggaatttttcctgaattatgcacccacagctg
ttcttagaagtatataaaagactaaaatgtaactagcagacatctatttgtgtagaagaagttaaggagggcagagaatataaatattaattaatattaa
ttctcagaaaatttcaaattcacattttaaacaagtaccagtgtttaggcagttttataatgttataattgataaaataaaacttattaaaatatgattt
catttgctgttcaaataatttctccattaacatgtgtattttctataataaaggaaaattacaaatcaaaaaaaaaaaaaaa
Pcdh15 exon 26
GTCTGTCTGTGATACCATGTTCATGGAGAACCCAGGTGTCAAAGTCTCTAGGACTGGAGCTAGGGGTGCCTGTGAGCCACAGTGTGGAATCTGGAACTAG
AACTGGGTCCTCTACAAGAGCAGCAAGTGTTCCTATCCACTGAgccatctctccggcatcttcctttggggttttaaaatccttaaaggagtcagtgagg
tcgcttagcaggaaagggcaattgctatcaatcctcaggacccatagttgagaagacttctgcattgtcctgtgattcctcattgctgtgacaaaacctc
agaggcacacacccaaggaattaaatgaatataatagaaatgtagtaaattgtaagcatttatgtatttggagaaaggtagaagaatccttaaactataa
acttatttggctaaacaaatagataaaaatgctagagaggaaaggtattatataacttattgtctaaaacatagacaaaatgttctgaaagaaaggcatt
gtgtaacctgtaaacacaacaaatgagaaaagttatttgaaaattgttaattatcacttttcattgaaacaaatagctttaaaagaaaaataatttactg
cactctcaaagtacaggatctggcatttccaaggactatctatgattttcagaaaagaaagatgataagttctctatacaaatataatacaggttattcc
cttccctctagtgtacgtgtaaatggaaagtaatagaagcttatgtggtgcccatgtatatgcttgtatatgccctggtaggaatgtagtgatgagactg
cctaacaaactcatgaattacatctcagaaatgcatggagcctaaagcatgtgtgcttttattcagtgcttgcttatttgaatattgctattaacatttc
atgactaacctttgccttctgtttaaaatgaagacacatgcatttccaacattgttccacttattaataactgttaatggcctgtaatcaattcctagtc
cattagctatgcaaataagtagcccattatgtaaattgaggttaattaattaattgtctaaagattttactctgctacaaaattagctactctatggatt
ttaactctgtcgcacatcatattaataaaagcaagaatctactcc
B
Il1rap
Long form variant
RQ (normalized to total)
1.5
1.0
0.5
0.0
*
0
5
20
40
40 cont.
U1
AMO conc. (uM)
Pcdh15
Long form variant
RQ (normalized to total)
1.5
1.0
0.5
0.0
0
5
20
40
40 cont.
U1
AMO conc. (uM)
C
Khdrbs1 (Sam68)
RQ (normalized to Gapdh)
1.5
1.0
0.5
0.0
n.s.
n.s.
0
20
40
U1
AMO conc. (uM)
D
U1 AMO conc.
0uM
20uM
PSD95
PSD95 /MAP2 /DAPI
E
PSD95 puncta
postsynaptic density (10² um²)
20
15
10
5
0
**
0uM
20uM
F
MAP2/DAPI⁺ cells
neuron density (10⁴ um²)
8
6
4
2
0
n.s.
0uM
20uM

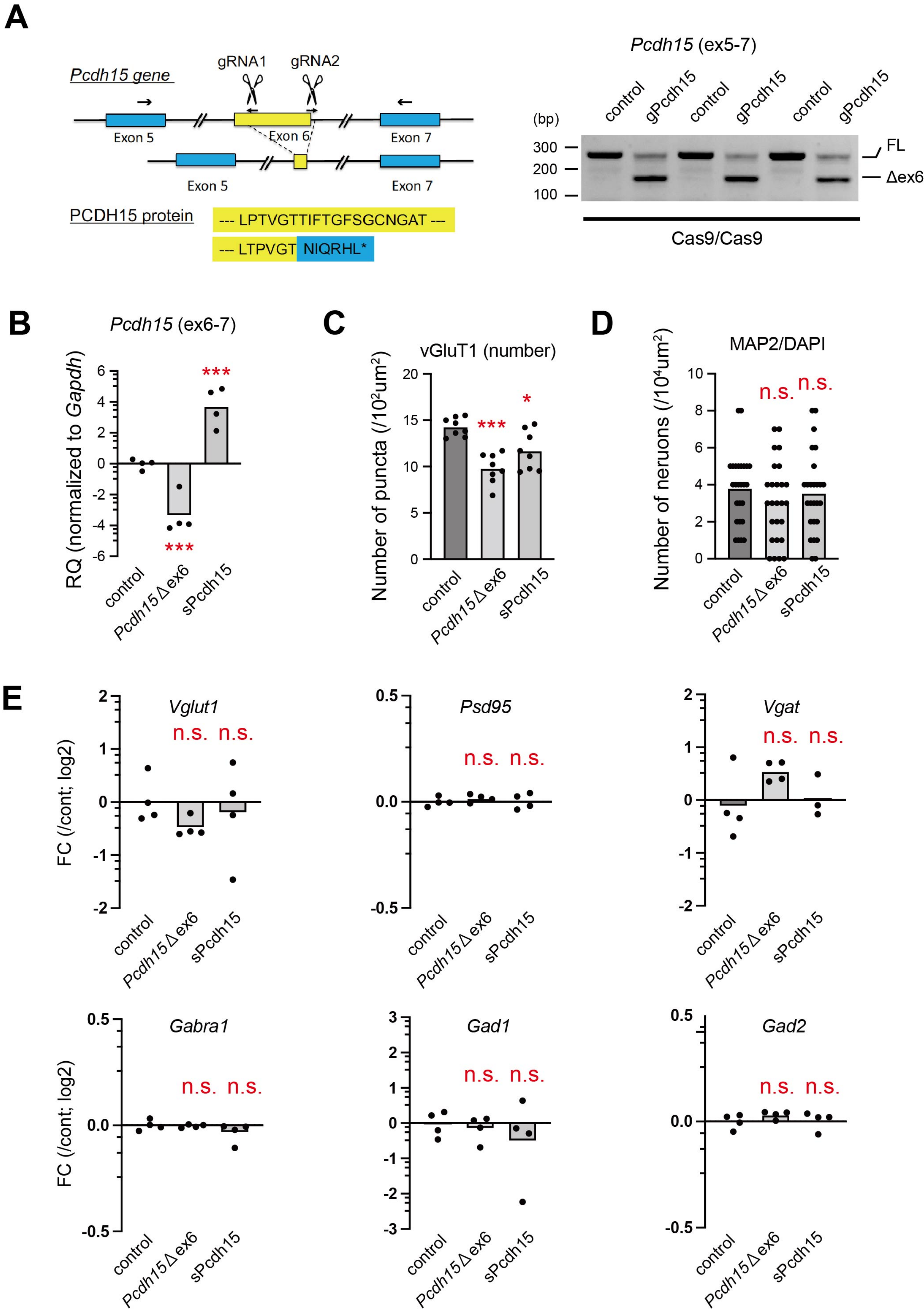
A
Pcdh15 gene
gRNA1
gRNA2
Exon 5
Exon 6
Exon 7
Exon 5
Exon 7
PCDH15 protein
--- LPTVGTTIFTGFSGCNGAT ---
--- LTPVGT NIQRHL*
Pcdh15 (ex5-7)
control
gPcdh15
(bp)
300
200
100
FL
Δex6
Cas9/Cas9
B
Pcdh15 (ex6-7)
RQ (normalized to Gapdh)
control
Pcdh15Δex6
sPcdh15
C
vGluT1 (number)
Number of puncta (/10²um²)
D
MAP2/DAPI
Number of neruons (/10⁴um²)
n.s.
E
Vglut1
Psd95
Vgat
Gabra1
Gad1
Gad2
FC (/cont; log2)
